# Therapeutic potential of rutin in premenstrual depression: evidence from *in vivo* and *in vitro* studies

**DOI:** 10.3389/fphar.2024.1525753

**Published:** 2025-01-14

**Authors:** Xiangjun Wang, Xiaowen Xia, Xianliang Song, Yi Zhou, Mingyu Ma, Yashuang Ren, Xitai Chen, Zenghui Xia, Yinghui Guo, Chunhong Song

**Affiliations:** ^1^ Laboratory of Traditional Chinese Medicine and Stress Injury of Shandong Province, Laboratory Animal Center, Central Hospital Affiliated to Shandong First Medical University, Jinan, China; ^2^ The Affiliated Taian City Central Hospital of Qingdao University, Taian, China; ^3^ Department of Pharmacy, Shandong Provincial Hospital Affiliated to Shandong First Medical University, Jinan, China; ^4^ School of Pharmacy, Shandong University of Traditional Chinese Medicine, Jinan, China; ^5^ Laboratory of Liver Viscera-State and Syndrome of Emotional Disease, College of Traditional Chinese Medicine, Shandong University of Traditional Chinese Medicine, Jinan, China

**Keywords:** rutin, premenstrual dysphoric disorder, depression, GABA_A_ receptors, neuronal damage, PMDD-depressed subtype model rats

## Abstract

**Introduction:**

Premenstrual dysphoric disorder (PMDD) is a cyclical mood disorder that severely affects the daily life of women of reproductive age. Most of the medications being used clinically have limitations such as low efficacy, side effects, and high cost, so there is an urgent need to discover safer and more effective medications. Rutin is a natural flavonol glycoside with various pharmacological properties including antidepressant. The study of the efficacy and mechanism of action of rutin in PMDD-depressed subtype model rats plays an important role in the discovery of new drugs for the treatment of PMDD.

**Methods:**

Binding of rutin to gamma-aminobutyric acid type A receptors (GABA_A_ receptors) was probed using molecular docking, microscale thermophoresis, radioactive receptor ligand binding assay and cell membrane clamp experiment. Behavioral tests in mice were performed to screen the optimal dose of rutin. Behavioral tests were performed to evaluate the effects of rutin on depressed mood, memory impairment, and social impairment in PMDD-depressed subtype model rats. HE staining and Golgi staining were performed to observe the neuronal damage in rat hippocampus. UHPLC-MS/MS targeted metabolomics was performed to detect the changes of neurotransmitter content in rat hippocampus. PCR array to detect the effect of rutin on mRNA expression of GABA_A_ receptor partial subunits in rat hippocampus.

**Results:**

The docking score of rutin with the GABA_A_ receptor benzodiazepine site was −11.442 and the gliding score was −11.470. The Kd of rutin with the GABA_A_ receptor (α1β2γ2) was 1.17 ± 0.89 μM. Rutin competed with [H^3^]-flunitrazepam for the GABA_A_ receptor benzodiazepine site and inhibited the inward flow of chloride ions (*P <* 0.05). In PMDD-depressed subtype rats, rutin alleviated depressed mood, memory impairment and social impairment, ameliorated hippocampal neuronal damage and reduces gamma-aminobutyric acid (GABA) and acetylcholine (ACh) levels (*P <* 0.05). Moreover, we found that rutin did not affect the relative mRNA expression of GABA_A_ receptor subunits in rat hippocampus.

**Discussion:**

Overall, rutin alleviated depressed mood, memory impairment and social impairment in PMDD-depressed subtype rats, which may be related to binding to GABA_A_ receptor benzodiazepine sites, inhibiting chloride ions inward flow, ameliorating hippocampal neuronal damage and reducing GABA and ACh levels. The results of this study provide an experimental basis and scientific evidence for the development of new drugs for the treatment of PMDD.

## 1 Introduction

Premenstrual dysphoric disorder (PMDD) is a cyclical mood disorder, with typical symptoms including anxiety and depression. Symptoms often appear in the late luteal phase 1 ∼ 2 weeks prior to menstruation, with symptoms resolving or disappearing after menstruation (Hantsoo and Payne, 2023). The U.S. Diagnostic and Statistical Manual of Mental Disorders, Fifth Edition, categorizes it as a severe type of PMS, and about 5% of women of childbearing age are affected by PMDD (Lanza Di Scalea and Pearlstein, 2019). Chinese epidemiological surveys have shown that 27.5% of women with PMDD exhibit a “depressive” type (Qiao et al., 2017; Xue et al., 2021), which severely affects their socialization, work, and quality of life. FDA-approved agents for PMDD include selective serotonin reuptake inhibitors (fluoxetine and sertraline) and oral contraceptives (drospirenone/ethinyl estradiol), but 40% of women are still resistant to this drugs, and some patients experience adverse effects such as insomnia and loss of appetite after taking them (Naguy et al., 2022). Finding new strategies for the safe and effective treatment of PMDD is a pressing research challenge.

The pathogenesis of PMDD is unclear, but studies have shown that its pathogenesis is closely related to neurotransmitters, ovarian hormone fluctuations, and the GABAergic system, among others (Re Nappi and Nappi, 2022). Among these, gamma-aminobutyric acid type A receptor (GABA_A_ receptor) expression and function are important factors in the etiology of mood disorders (Barki and Xue, 2022). In the late luteal phase, patients with PMDD have increased sensitivity of GABA_A_ receptors to the neurosteroid allopregnanolone content (Geng et al., 2024; Stiernman et al., 2023), and the concentration of gamma-aminobutyric acid (GABA) is altered (Bäckström et al., 2011). GABA_A_ receptors are a class of pentameric gated ion channels consisting of 19 subunits: α (1–6), β (1–3), γ (1–3), δ, ε, π, ρ (1–3), and θ. The combination of α1, β2, and γ2 is one of the most ubiquitously present forms of GABA_A_ receptors in the brain, with the benzodiazepine site is located right at the interface of the α and γ subunits (α+/γ-) (Thompson, 2024; Wen et al., 2022). GABA_A_ receptors can mediate CNS inhibitory synaptic transmission by regulating the flow of chloride ions after binding to GABA or benzodiazepines, among others (Study and Barker, 1981). New PMDD therapies targeting the GABA_A_ receptor, such as the use of the 5α-reductase inhibitor dutasteride to block the conversion of progesterone to allopregnanolone to alleviate PMDD symptoms, are also a potential option for patients with PMDD (Martinez et al., 2016). However, the cost of such drugs is high and dutasteride is mainly used for the treatment of benign prostatic hyperplasia, with fewer studies on its safety and efficacy in the treatment of mood disorders in women. Therefore, it is crucial to develop safe, effective, and low-cost novel GABA_A_ receptor-selective modulators for the treatment of PMDD. In addition, the hippocampus is an important part of the limbic system of the brain, which is involved in the body’s emotional regulation, learning and memory and other physiological functions (Wei et al., 2018). It has been found that resting-state functional connectivity is increased between L-hippocampus and R-frontal cortex and decreased between R-hippocampus and R-premotor cortex in women with comorbid bipolar disorder and PMDD (Syan et al., 2018). Gray matter density was significantly increased in the hippocampal cortex and decreased in the parahippocampal cortex in women with PMDD compared to healthy women (Jeong et al., 2012). The α1β2γ2 receptor is present in most brain areas and it is localized to interneurones in hippocampus and cortex, and cerebral Purkinje cells (Mckernan and Whiting, 1996). This suggests that the hippocampus is a potential neuroimaging target for PMDD.

Rutin is a flavonol glycoside widely found in natural drugs like *Ruta graveolens* and *Flos Sophorae Immaturus*, consisting of quercetin and rutinose (Budzynska et al., 2019), and it has various pharmacological actions like antioxidant and anti-inflammation (Ferreira et al., 2021). The specificity of their structures (different arrangements of hydroxyl, methoxy, and other substituents on the parent nucleus) makes many flavonoids, including rutin, have antidepressant and anxiolytic effects (Ko et al., 2020). An increasing number of researchers have found the benefits of rutin in neurological illnesses. For example, rutin exerted neuroprotective effects in chronic stress animal model (Parashar et al., 2017), modulated basolateral amygdala GABA_A_ receptors chloride channel to exert anxiolytic effects (Hernandez-Leon et al., 2017), exhibited antidepressant effects and inhibited acetylcholinesterase activity in a reserpine induced depression model (Foudah et al., 2022), and may exert anticonvulsant effects by binding to a GABA_A_–benzodiazepine receptor complex (Nassiri-Asl et al., 2008). Although rutin has been widely examined in psychiatric diseases, investigations on the mechanism of rutin’s deeper intervention and its involvement in the treatment of PMDD have not yet been published. We reviewed the literature and found that rutin has antidepressant effects and binds to the GABA_A_ receptor, which is a key target in the pathogenesis of PMDD. Therefore, we hypothesized that rutin may play a key role in the treatment of the depressed subtype of PMDD by affecting the GABA_A_ receptor.

Therefore, the aim of this study was to explore the efficacy and mechanism of action of rutin on PMDD based on GABA_A_ receptors through *in vivo* and *in vitro* studies. Through molecular docking, microscale thermophoresis, radioactive receptor ligand binding assay and cell membrane clamp experiment, it was demonstrated that rutin could bind to GABA_A_ receptor and inhibit chloride ion inward flow. Behavioral tests in mice were performed to screen the optimal dose of rutin. Through behavioral tests, we also found that rutin alleviated depressed mood, memory impairment and social impairment in PMDD-depressed subtype model rats. Rutin was found to ameliorate hippocampal neuronal damage in rats by HE staining and Golgi staining. Using UHPLC-MS/MS targeted metabolomics to detect changes of neurotransmitter content in rat hippocampus revealed that rutin affected the levels of GABA and ACh contents in rat hippocampus. PCR array found that rutin did not affect mRNA expression of GABA_A_ receptor partial subunits in rat hippocampus. This study provides an experimental basis for the development of new drugs for the treatment of PMDD.

## 2 Materials and methods

### 2.1 Molecular docking

The hydrogen bond donor, the hydrogen bond acceptor, the P-gp substrate, and the PAINS alert were calculated by the online server Swiss-ADME (Daina et al., 2017). LigPrep in the Schrodinger 2018 (Schrödinger, 2018-1, NY, United States) suite was used to process target compounds. OPLS_2005 force field was selected, and pH was set to 7 ± 2. The remaining parameters were kept as default. Subsequently, GABA_A_ receptor (PDB ID: 6D6T) was loaded, and the protein preparation wizard was used to perform pretreatments like deleting water, hydrogenating, and charging (Zhou et al., 2020). Next, the amino acids near binding sites (GABA site or Benzodiazepine site) in the processed protein were chosen to build a docking grid with 20 Å, and the remaining parameters were kept as default. The glide module in the Schrodinger 2018 suite was used to dock the processed ligands and the docking grid. The docking accuracy was XP mode, and the remaining options remained the default (Friesner et al., 2006). The conformation with the highest docking score was analyzed in the bonding mode and studied further in subsequent dynamic simulations. The figures were plotted with Pymol (Schrödinger, v.3.0.3, NY, United States) and Ligplot plus (Roman Laskowaski, v2.2.9, Cambridge, UK) (Laskowski and Swindells, 2011).

### 2.2 Microscale thermophoresis (MST)

pcDNA3.1-GABRA1, pIRES2-EGFP/GABRB2, pcDNA3.1-GABRG2, pcDNA3.1-GABARAP were purchased from Research Cloud Bio (Shandong, China). The above plasmids were amplified using DH5αE. coli receptor cells (Takara, 9057, Beijing, China). After that experimental HEK293 cells were transiently infected according to the instructions of Lipofectamine 3000 kit (Thermo Fisher, L3000008, Shanghai, China) according to the cDNA ratio of α1, β2/EGFP, γ2 and AP as α:β/EGFP: γ2:AP = 1:1:2:1. The green fluorescence intensity was observed under a fluorescence microscope 48 h after transfection, while the protein was extracted for quantification afterwards. Rutin (GUANGRUN biotechnology, GH-136-190429, Jiangsu, China) and betulinic acid (Shanghai yuanye Bio-Technology Co., Ltd, S31419, Shanghai, China) were diluted with DMSO into 16 solutions with different concentration gradients, after which it was added to the protein solution separately. The reacted samples were aspirated by capillary tubes and assayed on the MST machine (NanoTemper Technologies, Monolith, Munich, Germany). The data were analyzed using MO.Affinity Analysis v2.3 software (NanoTemper, v.2.3, Munich, Germany). Where the signal to noise ratio higher than 5 was considered to be significantly different (Huang and Zhang, 2021). Lower Kd values indicated stronger binding of rutin to the GABA_A_ receptor (Liu et al., 2023). The data from the three replicate measurements were combined in the experiment for error point removal and finally a free fit of the affinity curve was performed (Chebib and Schwab, 2021).

### 2.3 Radioactive receptor ligand binding assay

Rat whole brain was weighed and homogenized in 20 times pre-cooled sucrose solution (0.32 M), centrifuged at 4°C and 1,000 g for 10 min. The supernatant was centrifuged at 4°C and 20,000 g for 20 min, and the precipitate was dissolved in 50 mM Tris-HCl to bring the protein concentration to 0.8–1.6 mg/mL and placed at −80°C for storage (Vogel). For formal experiments, the reaction solution was mixed in the following proportions: (1) Total binding tube: 48 µL membrane receptor + 1 µL DMSO + 1 µL [H^3^]-flunitrazepam (PerkinElmer, 2461442, MA, United States); (2) Non-specific binding tube: 48 µL membrane receptor + 1 µL diazepam (10^−6^ M) (Sigma, D-907, MO, United States) + 1 µL [H^3^]-flunitrazepam; (3) Rutin binding tube: 48 µL membrane receptor + 1 µL rutin (Shanghai yuanye Bio-Technology Co., Ltd., Y16M9S61523, Shanghai, China) + 1 µL [H^3^]-flunitrazepam. The above tubes were incubated in a shaker at 37°C for 2 h immediately after adding [H^3^]-flunitrazepam. The solution in the filter plate was withdrawn on glass fiber filter paper, dried for 0.5 h, and measured by adding scintillation solution (PerkinElmer, 6013591, MA, United States) and counted by XH-6925 liquid flash meter (Middendorp et al., 2015; Vogel, 1986). The inhibition rate of each compound on receptor-ligand binding was calculated using the following formula: Inhibition rate = (total binding tube cpm - rutin binding cpm)/(total binding tube cpm - non-specific binding tube) × 100%.

### 2.4 Cell membrane clamp experiment

The hippocampus of neonatal rats was separated, and 2 mL of 0.25% trypsin-EDTA digestion solution was added and put into a 37°C water bath for 5 min digestion. The digestion was then terminated by adding DMEM + 20% FBS + 1% Penicillin-Streptomycin medium, filtered through a 70 µm sieve into a centrifuge tube, and centrifuged at 800 rpm for 5 min. The supernatant was discarded. Then, 5–10 mL of media was added, and a single-cell suspension was created by gently blowing. The cell density was adjusted at 1 × 10^6^/mL into a culture dish containing slides and incubated in a 37°C/5% CO_2_ cell culture chamber. After 7 days of incubation, use for electrophysiological experiments.

Rutin was dissolved in DMSO (300 mM), and diluted with extracellular solution (NaCl 125 mM; MgCl_2_ 2 mM; KCl 2 mM; NaHCO_3_ 26 mM; KH_2_PO_4_ 1.25 mM; CaCl_2_ 2 mM; Glucose 10 mM, pH adjusted to 7.4 with NaOH) in gradients of 100 μM, 300 μM, 1,000 µM. Bicuculline (Selleck, S7071, TX, United States), a positive control drug, was diluted to 30 µM with an extracellular solution. GABA_A_-type receptor channel currents were recorded using a whole-cell aspiration breakthrough membrane clamp technique. The borosilicate glass microelectrode tip resistance was 2–6 MΩ. The membrane potential was clamped at −60 mV in Gap-free mode and noted in extracellular fluid for 1 min. When the GABA_A_ current amplitude shrank and desensitized after paracellular perfusion administration, drug action was considered to reach a steady state (Bian et al., 2019). In the experiment, the membrane resistance was greater than 1,000 MΩ, and the leakage current was less than 10% of the ion channel current to meet the detection criteria. The inhibition rate of GABA_A_ receptor current by the compound to be tested was calculated based on the following equation: inhibition rate = (1-A_2_/A_1_) × 100%, where A1 represents the average of the peak current before the compound was added, and A2 represents the average of the peak current following the compound’s addition.

### 2.5 Animals

100 SPF-grade CD-1 male mice (18–20 g) and 100 SPF-grade Wistar female rats (170–200 g) were purchased from Beijing Vital River Laboratory Animal Technology Co., Ltd [SCXK (Beijing) 2016–0006]. The laboratory settings were reversed day and night, with lights on at 20:00 and off at 8:00 daily, temperature 23°C ± 1°C, and humidity 50% ± 10% RH. Five animals per cage were housed in IVC cages and fed SPF-grade rat-mouse maintenance chow (Beijing Keao Xieli Feed Co., Ltd). The animals were kept for 7 days before participating in the formal test. All experiments were approved by the Experimental Animal Welfare Ethics Review Committee of the Laboratory Animal Center of the Central Hospital Affiliated to Shandong First Medical University (JNCHIACUC2021-81, approval date: 18 October 2021) and were conducted following the ARRIVE guidelines.

### 2.6 Animal experimental design

#### 2.6.1 Rutin dose screening experiment

**FIGURE 1:**
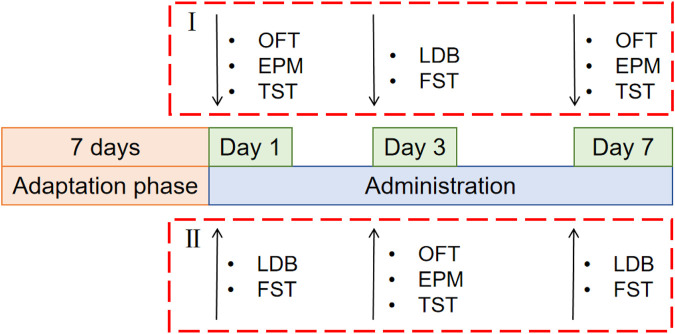
Schematic diagram of the experimental schedule for screening the optimal dose of rutin. 100 mice were randomly distributed into two parts (I and II), each of which was randomly divided into the following five groups: control group, fluoxetine group (4.05 mg/kg), rutin high dose group (50 mg/kg), rutin medium dose group (25 mg/kg), rutin low dose group (12.5 mg/kg), n = 10. The open field test (OFT), elevated plus maze (EPM), tail suspension test (TST), light/dark box (LDB) and forced swimming test (FST) were performed after single, consecutive 3 days and consecutive 7 days administrations.

The test was conducted in two batches, and 100 mice were randomly distributed into two parts (I and II), each of which was randomly divided into the following five groups: control group, fluoxetine group (Lilly Suzhou Pharmaceutical, J20160029, Jiangsu, China) (4.05 mg/kg), rutin high dose group (GUANGRUN biotechnology, GH-136-190429, Jiangsu, China) (Rutin H, 50 mg/kg), rutin medium dose group (Rutin M, 25 mg/kg), rutin low dose group (Rutin L, 12.5 mg/kg) (n = 10). Mice were gavaged daily at 10 mL/kg and behavioral tests were performed on days 1, 3, and 7 (Figure 1).

#### 2.6.2 Preparation and administration of PMDD-depressed subtype model rats

**FIGURE 2:**
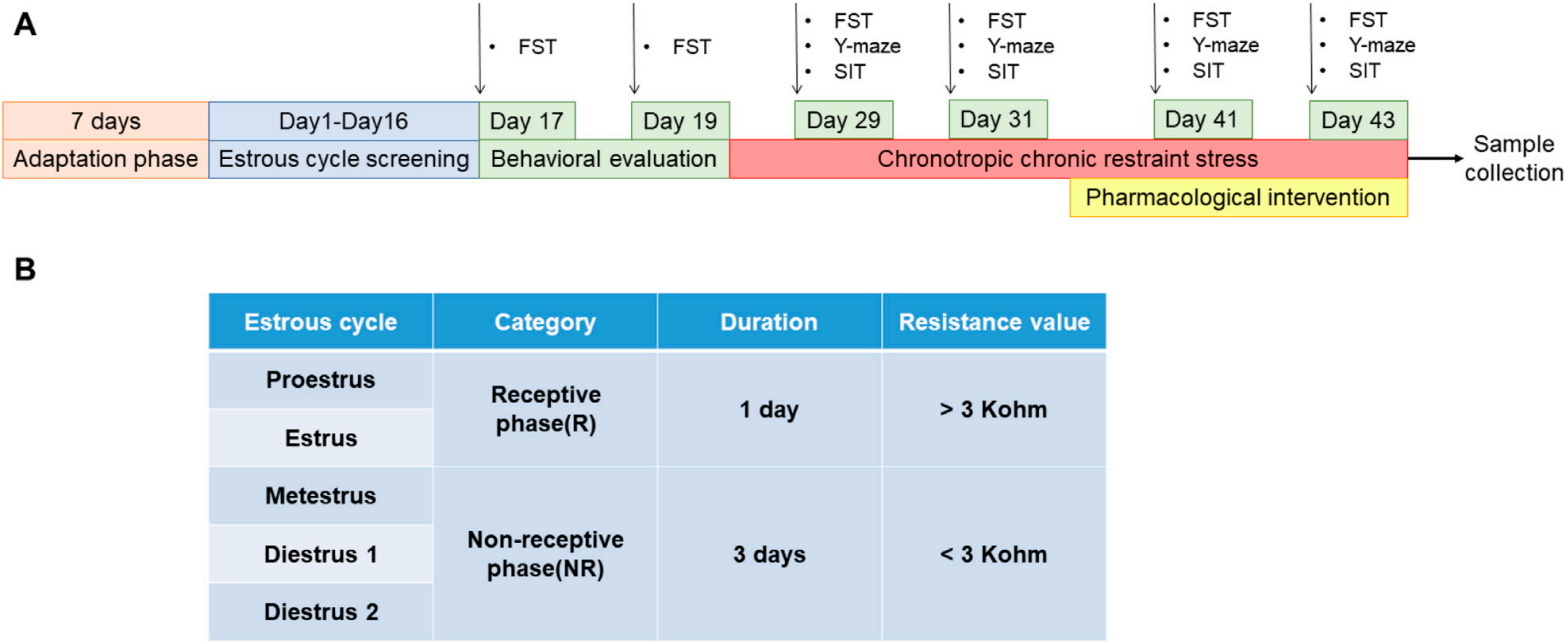
Preparation and administration of PMDD-depressed subtype model rats. **(A)** Experimental schedule of rutin intervention in PMDD-depressed subtype rats, and **(B)** corresponding table of the time and vaginal resistance values of each estrous cycle stage in rats. This study was designed with 4 groups including control group, model group, fluoxetine group (2.7 mg/kg) and rutin group (8.65 mg/kg), n = 10. The forced swimming test (FST), Y-maze and social interaction testing (SIT) were performed before and after the administration in rats.

The estrous cycle in rats includes proestrus, estrus, metestrus, and diestrus 1 and 2 (Hubscher et al., 2009; Wei et al., 2020). The proestrus and estrus are the rats' receptive phases (R). The metestrus, diestrus 1 and 2 are the non-receptive phases (NR) of the rats (Li et al., 2023) (Figure 2B). The rats' electrical resistance values were measured daily from 13:00 to 14:00 using an electronic monitor of the vaginal estrous cycle (Muromachi, MK-11, Tokyo, Japan) for 16 days. Rats with at least two consecutive regular estrous cycles (4 days per cycle) (Gao et al., 2022a) were selected for FST experiments during their NR and R phases. The difference is sorted from largest to smallest by the NR phase immobility time minus the R phase immobility time. Ten rats with a difference near zero were screened out and assigned to the control group, while the remaining rats were exposed to chronically bound stress at specific times to generate PMDD-depressed subtype rats. The model rats were restrained for 6 h daily (14:00–20:00) during the NR phases, and the rest of the time was free. After binding for two estrous cycles, rats were submitted to behavioral tests during the NR and R phases of the third estrous cycle. The difference is sorted from largest to smallest by the NR phase immobility time minus the R phase immobility time (Wei et al., 2020). The top 30 rats of the sort were included in the experimental group, and they were randomly divided into the following three groups: model group, fluoxetine group (2.7 mg/kg), and rutin group (8.65 mg/kg) (n = 10). After 8 days of continuous gavage, behavioral tests were performed during the NR and R phases of the 3rd estrous cycle (Figure 2A).

### 2.7 Behavioral testing

All tests were performed during the dark part of the inverted cycle under dim red light (30 lux) (14:00–17:00 daily) (Hernandez-Leon et al., 2017).

Open field test (OFT): The mice were placed in the central area of a 50 × 50 cm open field box (Shanghai Xinsoft, XR-XZ301, Shanghai, China), total distance during 6 min was collected using animal behavior analysis software (Shanghai Xinsoft, SuperMaze+, Shanghai, China) (Wang et al., 2021; Seibenhener and Wooten, 2015).

Elevated plus maze (EPM): The mice elevated plus maze (Shanghai Xinsoft, XR-XG201, Shanghai, China) consists of two opposing 35 × 5 × 10 cm closed arms and two opposing 35 × 5 cm open arms with a 5 × 5 cm open sections in the center. The mice were placed in the central area, and open arm entry times (OE), closed arm entry times (CE), time in the open arm (OT), and time in the closed arm (CT) were recorded within 5 min using SuperMaze+. The OE% and OT% were calculated from the above data: OE% = OE/(OE + CE) × 100%, OT% = OT/(OT + CT) × 100% (Green et al., 2021; Lin et al., 2021).

Tail suspension test (TST): Mice were inverted at the top of the TST box (30 × 30 × 50 cm, Shanghai Xinsoft, XR-XQ202, Shanghai, China). After mice were acclimatized for 2 min, the immobility time within 4 min was recorded using SuperMaze+ (Liu et al., 2022; Liu H. et al., 2018).

Light/dark box (LDB): The light/dark box (Shanghai Xinsoft, XR-XB110, Shanghai, China) consists of two 13 × 16 × 20 cm compartments with a door between the two boxes that allow the mice to pass freely. Above the light box, an incandescent bulb was on, and above the dark box, a red lamp was on. The mice were placed in the center of the light box, and SuperMaze + recorded the number of cases through the box and the time spent in a light box within 5 min (Borbély et al., 2017; Li et al., 2021).

Forced swimming test (FST): The day before the experiment, mice were placed in a transparent cylinder (25 cm in height, 15 cm in diameter, 20 cm depth of water with temperature of 23–25°C) for 6 min of pre-swimming. The size of the transparent cylinder in the rat FST is 40 cm in height, 20 cm in diameter, and 30 cm in water depth. During the formal experiment, the cylinder was placed in the FST chamber (Shanghai Xinsoft, XR-XQ202, Shanghai, China), and mice/rats were slowly put into the water. After mice/rats were acclimatized for 2 min, the immobility time within 4 min was recorded using SuperMaze+ (Borbély et al., 2017).

Y-maze: The Y-maze chamber (Shanghai Xinsoft, XR-XY103, Shanghai, China) consisted of three rectangular bodies of 40 × 10 × 25 cm with an angle of 120° to each other. The rats were placed at the end of either arm of the Y-maze, and SuperMaze+ was used to collect the following within 8 min: (1) the total number of arm entries: the number of times the rat entered all four feet into the arm; (2) the maximum number of rotations: total number of arm entries-2; (3) number of rotations: the number of consecutive entries into the three arms and finally the spontaneous behavior score was estimated (spontaneous rotation score) = number of rotations/maximum number of rotations × 100% (Lamtai et al., 2021; Okojie and Oyekunle, 2014).

Social interaction testing (SIT): the social box (Shanghai Xinsoft, XR-XJ117, Shanghai, China) consisted of three 54.6 × 26 cm compartments with a door between each compartment allowing the rat to pass freely, and a wire cage (20 cm in diameter and 18 cm in height) in each corner of the left and right compartments. A day prior, rats were exposed to the social box for 5 min to acclimatize. For the formal test, a female rat of the same age that was unfamiliar to the experimental rat was placed into the iron cage of the left box. A toy was put into the iron cage of the right box, and the experimental rat was placed into the middle box again to test for 5 min. The time and times of contact with the unfamiliar rat were recorded with SuperMaze+ (Li et al., 2023).

### 2.8 Hematoxylin-eosin staining

After rats were anesthetized with 2% sodium pentobarbital, the heart was perfused with 4% paraformaldehyde. When the liver of the rats turned white, the whole brain was isolated. The isolated brain tissue was fixed in 4% paraformaldehyde at 4°C for 24 h. Afterwards, the tissue was rinsed slowly using running water for 3 h to remove paraformaldehyde. The tissue was dehydrated using gradient ethanol (75% ethanol for 2 h, 85% ethanol for 2 h, 90% ethanol for 1.5 h, 95% ethanol for 1.5 h, 100% ethanol for 30 min, and 100% ethanol for 30 min) and then immersed in clearing agent (Tianxing Linhua, TX2192500, Guangxi, China) for 3 times for 15 min each time. The tissue was soaked in the wax solution for 3 h and then embedded using an embedding machine (Yaguang pathology, YB-6LF, Hubei, China). The pre-cooled wax blocks were sliced using a slicer (Leica microsystems, RM2255, Shanghai, China) with the thickness set to 5 μm. The slices were placed in water at 42°C for spreading, and then placed on a dryer (Leica microsystems, HI1220, Shanghai, China) at 65°C for 2 h. Sections were processed using an HE staining kit (Bioss, C02-04004, Beijing, China). The sections were deparaffinized in clearing agent for 5 min, repeated twice, 100% ethanol for 5 min, 90% ethanol for 2 min, 70% ethanol for 2 min, and distilled water for 2 min. After hematoxylin staining for 5 min, the sections were rinsed with running water and then stained with 70% ethanol solution containing 0.5% hydrochloric acid. After rinsing with running water, the sections were immersed in 1% eosin aqueous solution for 5 min. After the sections were washed with distilled water to remove the staining solution attached to the slides, they were subjected to 70%, 80%, 90%, and 95% ethanol and twice 100% ethanol for dehydration, and twice clearing agent for 2 min each. The sections were sealed in sealer (Jiangyuan, JY2182050, Jiangsu, China) and photographed under a Zeiss microscope (Axio Imager.A2, Oberkochen, German). Three rats were in each group, and 3 sections of each rat tissue were taken, and 1 image was obtained from each tissue section. And they were analyzed using SlideViewer software (3DHISTECH, v.2.5.0.143918, Budapest, Hungary) (Liang et al., 2023).

### 2.9 Golgi staining

Whole brains were isolated after rats were deeply anesthetized with 2% sodium pentobarbital. The tissues were stained according to the instructions of the FD rapid golgistintm kit (FD NeuroTechnologies, PK401, MD, Columbia). The tissues were immersed in an equal volume mixture of solutions A and B configured 24 h in advance, replaced with a new staining solution the following day, and stored in the dark environment for 2 weeks before transferring the brain tissues to solution C. The solution was replaced 24 h later and stored in the dark at room temperature for 72 h. After draining the surface liquid, the tissue was cut into 100 μm slices using a vibrating microtome (Campden instruments, 7000smz-2, Loughborough, UK). Sections were rinsed twice with Milli-Q water for 4 min each, placed in a mixture of solution D:E:Milli-Q water = 1:1:2 for 10 min, rinsed twice with Milli-Q water for 4 min each, dehydrated in 50%, 75%, and 95% ethanol for 4 min each, and dehydrated in anhydrous ethanol for 4 times for 4 min each time. Slices were cut in clearing agent for 3 times for 4 min each and were sealed with a sealer. Pyramidal neurons in the hippocampus were photographed using an Olympus BX530 (Tokyo, Japan) light microscope with a field of view of 1,000×. Neuronal dendritic spine density was estimated using ImageJ (Version 1.52a, Wayne Rasband National Institutes of Health, United States) (Du, 2019). Three rats were in each group, and 2 sections of each rat tissue were taken, and 1 image was obtained from each tissue section.

### 2.10 Ultra-high performance liquid chromatography-MS/MS (UHPLC-MS/MS) targeted metabolomics

The hippocampus was isolated after anesthetizing rats with 2% pentobarbital sodium and the following neurotransmitter levels were determined: serotonin (5-HT, Sigma, 14,927, MO, United States), acetylcholine (Ach, Sigma, A6625, MO, United States), epinephrine (E, Sigma, E-143, MO, United States), norepinephrine (NE, Solarbio, SN8550, Beijing, China), dopamine (DA, Sigma, H8502, MO, United States), glutamate (Glu, Sigma, G1251, MO, United States), gamma-aminobutyric acid (GABA, Sigma, A2129, MO, United States). The standard solution was obtained by diluting 20 µL of 1 mmol/L of each neurotransmitter standard with 140 µL of 80% acetonitrile (0.1% formic acid). 80 μL of standard solution was added to 40 µL of 100 mmol/L sodium carbonate solution and 40 µL of 2% benzoyl chloride acetonitrile solution and derivatized for 30 min at room temperature to obtain the external standard. 80 μL of standard solution was added to 40 µL of 100 mmol/L sodium carbonate solution and 40 µL of 2% benzoyl chloride-D5 acetonitrile solution and derivatized for 30 min at room temperature to obtain the internal standard. Stabilize 320 µL of the internal standard with 1,680 µL of 65% acetonitrile (0.1% formic acid). The first point of the calibration curve was prepared by mixing 160 µL of the external standard with 10 µL of the internal standard, and then diluting the standard mixture by 50% up to 20 points. The standard mixture was centrifuged at 12,000 rpm for 5 min at 4°C, and 40 µL of the supernatant and 20 µL of water were removed for testing. Take 10 ± 0.5 mg of the hippocampal sample to be tested in 80 µL of extraction solution (acetonitrile containing 0.1% formic acid, pre-cooled at −20°C), add 20 µL of water, add the steel beads, and vortex for 30 s to mix. 40 Hz homogenization was done for 240 s, and sonication was performed in an ice-water bath for 5 min. Repeat homogenization and sonication steps three times. The samples were precipitated at −40°C overnight. After centrifugation at 4°C and 12,000 rpm for 15 min, 80 µL of the supernatant was taken and incubated with 40 µL of 100 mmol/L sodium carbonate and 40 µL of 2% benzoyl chloride for 30 min at room temperature, 10 µL of the internal standard was added, and then centrifuged at 4°C and 12,000 rpm for 15 min, 40 µL of the supernatant and 20 µL of the supernatant were taken and assayed on the water machine. In this project, the target compounds were separated by liquid chromatography on a Waters ACQUITY UPLC HSS T3 (100 × 2.1 mm, 1.8 µm) column using a SCIEX ExionLC ultra-performance liquid chromatography(SCIEX, SCIEX ExionLC, MA, United States). The A phase of the liquid chromatography was 0.1% formic acid and 1 mM ammonium formate aqueous solution, and the B phase was acetonitrile. The column chamber temperature was 40°C, the sample tray was set at 6°C, and the injection volume was 2 µL. For data acquisition, mass spectrometry (SCIEX, AB Sciex QTrap 6500+, MA, United States) was performed in multiple reaction monitoring (MRM) modes. Ion source parameters were as follows: IonSpray Voltage: +5000 V, Curtain Gas: 35 psi, Temperature: 400°C, Ion Source Gas 1:60 psi, Ion Source Gas 2:60 psi (Wong et al., 2016; Liu R. et al., 2018).

### 2.11 PCR array

Samples were homogenized for 1 ∼ 2 min after adding 1 mL Trizol and grinding beads until no obvious tissue mass was observed by the naked eye. Centrifuge at 12000 g for 5 min at 4°C and retain the supernatant. Add chloroform according to 1:5 (chloroform:Trizol), up and down for 30 s, leave at room temperature for 5 min. 4°C, centrifuge at 12000 g for 15 min. Pipette the upper layer of aqueous phase in a new centrifuge tube, add an equal volume of isopropanol, up and down, precipitate at room temperature for 10 min. 4°C, centrifugation at 12,000 g for 10 min, discard the supernatant, add 1 mL of 75% ethanol to wash the precipitate. 7,500 g, centrifugation at 4°C for 5 min. Discard the supernatant and retain the precipitate. Dry the sample at room temperature for 5 min, and dissolve the RNA sample with appropriate amount of DEPC-treated water. Rat hippocampal RNA was reverse transcribed to cDNA using the WCGENE mRNA cDNA Synthesis Kit (WCGENE, WC-SJH0001, Shanghai, China). cDNA was mixed with Wcgene^®^ mRNA qPCR mix (WCGENE, WC-SJH0002, Shanghai, China) and added to a customized PCR array 96-well plate for qRT-PCR (Table 1). The raw Ct values of all genes were analyzed, and the fold change was calculated using the 2^-ΔΔCt method for comparison. Firstly, we used the Ct value of the target gene minus the Ct value of the internal reference gene of each group of samples to get ΔCt. Then, we used the ΔCt of each target gene of each group of samples minus the ΔCt of the control group samples and take the opposite number of all the results at the same time to get −ΔΔCt. Finally, carry out a power of 2 operation on the −ΔΔCt, i.e., 2^∧−ΔΔCt^, and then arrive at the Fold Change. Genes with fold-changes more than or less than 2.0 were considered to be of biological significance (Yue et al., 2019; Livak and Schmittgen, 2001).

**TABLE 1:** PCR array target gene list.

	1	2	3	4	5	6	7	8	9	10	11	12
A	*Abat*	*Cacna1f*	*Cnr1*	*Gabbr1*	*Gabrb2*	*Gec1*	*Grm8*	*Htr2c*	*Maoa*	*Pla2g2a*	*Rapgef3*	*Syngap1*
B	*Abhd6*	*Cacna1g*	*Cnr2*	*Gabbr2*	*Gabrb3*	*Gls*	*Hap1*	*Htr3a*	*Map2k1*	*Plcg1*	*Slc12a5*	*Tph*
C	*Adcy1*	*Cacna1h*	*Creb1*	*Gabra1*	*Gabrd*	*Glul*	*Htr1a*	*Htr4*	*Mapk1*	*Plcl11*	*Slc18a1*	
D	*Cacna1a*	*Cacna1i*	*Daglb*	*Gabra2*	*Gabrg1*	*Gnaq*	*Htr1b*	*Htr5a*	*Mapk8*	*Pld1*	*Slc6a1*	
E	*Cacna1b*	*Cacna1s*	*Ddc*	*Gabra3*	*Gabrg2*	*Gphn*	*Htr1d*	*Htr6*	*Mgll*	*Prkaca*	*Slc6a12*	
F	*Cacna1c*	*Camk2a*	*Dusp1*	*Gabra4*	*Gabrg3*	*Gria1*	*Htr1f*	*Htr7*	*Napepld*	*Prkcg*	*Slc6a4*	
G	*Cacna1d*	*Camk4*	*Ephb1*	*Gabra5*	*Gabrr1*	*Grin1*	*Htr2a*	*Itpr1*	*Nlgn2*	*Prkg1*	*Src*	
H	*Cacna1e*	*Casp3*	*Gabarapl2*	*Gabrb1*	*Gad1*	*Grin2b*	*Htr2b*	*Kcnj3*	*Nrxn1*	*Raf*	*Syn1*	

### 2.12 Statistical analysis

Statistics were performed using GraphPad Prism 8 analysis software (GraphPad, v.8.3.0, MA, United States), and data were expressed as Mean ± SD. When comparing the two groups of samples, normality of the data distribution was tested using the Shapiro-Wilk test, and homogeneity of variance was compared using the F test. Parametric tests and unpaired t-tests are used when the data were normally distributed and satisfied homogeneity of variance. Nonparametric tests and Mann-Whitney tests are used when the data are not normally distributed. When comparing more than four samples, the Shapiro-Wilk test was used to test the normality of the data distribution, and the Brown-Forsythe test was used to compare the homogeneity of variance. Ordinary one-way ANOVA and Tukey’s multiple comparisons test were used when the data were normally distributed and satisfied homogeneity of variance. Brown-Forsythe and Welch ANOVA tests and Dunnett’s T3 multiple comparisons test were used when the data were normally distributed but did not satisfy homogeneity of variance. Kruskal-Wallis test and Dunn’s multiple comparisons test were used when the data were not normally distributed. *P* < 0.05 was statistically significant.

## 3 Results

### 3.1 Rutin binds to GABA_A_ receptors and inhibits channel currents

**FIGURE 3:**
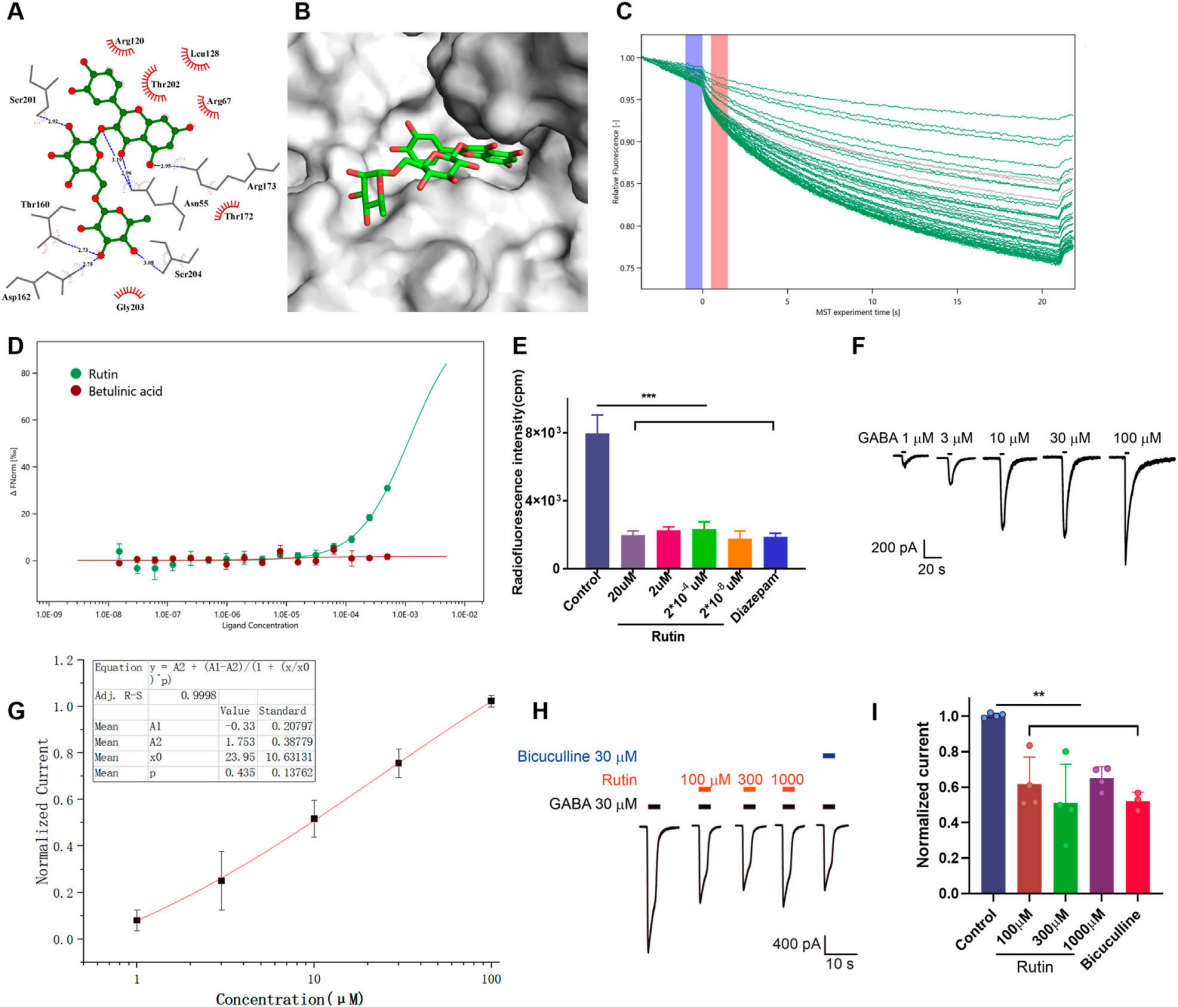
Rutin binds to GABA_A_ receptors and inhibits channel currents. **(A)** Schematic representation of the 2D interaction of the compound rutin with the active site residues of the target protein GABA_A_ receptor. **(B)** Schematic representation of the docking pocket of the GABA_A_ receptor with the 3D structure of rutin. **(C)** Thermophoresis curves obtained using rutin as ligand by microscale thermophoresis (MST). **(D)** MST analysis of the binding affinity of rutin for the GABA_A_ receptor. **(E)** Radioactivity intensity in rutin binding tubes at different concentrations (P = 0.0001), n = 2/group. **(F, G)** EC50 values and fitted curves of GABA on chloride channel currents. **(H, I)** Effect of simultaneous perfusion of rutin and GABA on chloride channel currents (*P* = 0.0006), n = 3 ∼ 4/group. Results are shown as Mean ± SD. One-way ANOVA analysis followed by Tukey’s multiple comparison test. **P < 0.01, ***P < 0.001.

First, Glide-based molecular docking was done to investigate if rutin can bind to the GABA_A_ receptor. The rutin’s docking score with the GABA site was −9.105, and the glide score was −9.133; the docking score with the benzodiazepine site was −11.442, and the glide score was −11.470. As shown in Figure 3A, rutin interacts with Arg173, Asn55, Asp162, Ser201, Ser204, and Thr160 residues. The conformational analysis of the molecular docking structure showed that rutin was in the docking pocket formed by the GABA_A_ receptor (Figure 3B). This results indicated that rutin had a strong binding ability to the benzodiazepine site of the GABA_A_ receptor. To further verify the binding of rutin to GABA_A_ receptors, we constructed recombinant GABA_A_ receptors (α1β2γ2) for MST analysis. The increase of rutin concentration significantly affected the thermophilic movement of GABA_A_ receptor and the Kd was 1.17 ± 0.89 μM, and combined with Figures 3C, D, we found that rutin binds better to GABA_A_ receptors (α1β2γ2) compared to betulinic acid. The rutin’s binding to the GABA_A_ receptor was later verified using a receptor-ligand binding assay. The results are shown in Figure 3E. The radio fluorescence intensity of all concentrations of rutin (2 × 10^−8^–20 µM) binding tubes was significantly lower than the control group, consistent with the results of non-specific binding tubes of diazepam. Rutin’s impact on GABA_A_ receptor channel currents was investigated using primary hippocampal neuronal cells grown in a cell membrane clamp. Sequential perfusion of 1/3/10/30/100 µM GABA could activate the GABA_A_-type receptor current in a gradient (Figure 3F), and the fitted curve showed that the EC50 was 23.95 ± 10.63 µM (Figure 3G). Thus, the current induced by 30 µM GABA was chosen as the control group in this experiment. Different doses of rutin (100/300/1,000 µM) were simultaneously perfused into neuronal cells with GABA. Moreover, the current evoked by GABA was significantly reduced (Figures 3H, I), indicating that rutin can bind to GABA_A_ receptors and inhibit Cl^−^ inward flow.

### 3.2 Rutin low dose improves depression in mice

**FIGURE 4:**
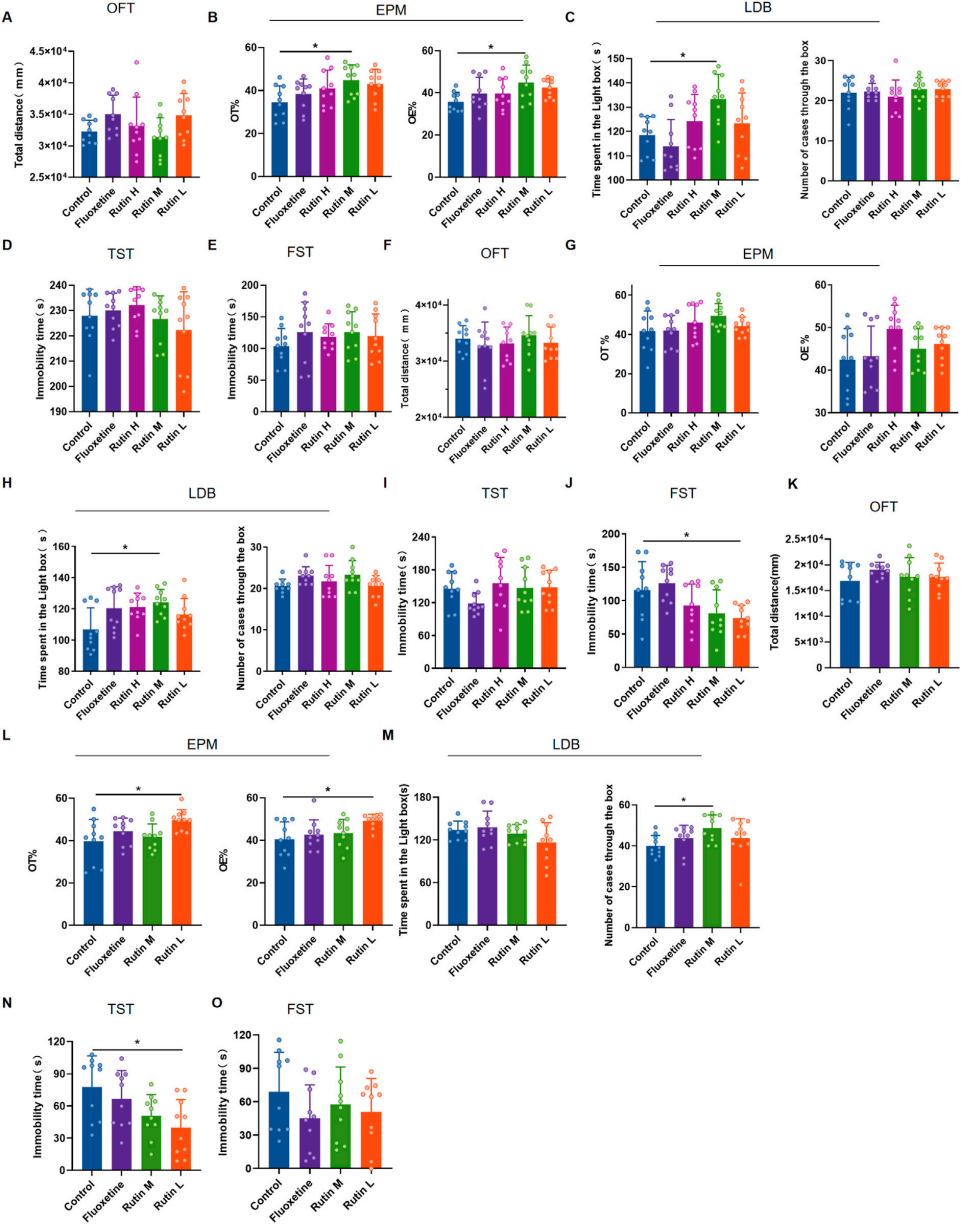
The behavioral experimental results of rutin administration in mice. **(A–E)** The behavioral experimental results of a single administration of rutin. **(A)** Total distance for open field test (OFT) (*P* = 0.0743); **(B)** OT% (*P* = 0.0291) and OE% (*P* = 0.0427) for elevated plus maze (EPM); **(C)** Time spent in the light box (*P* = 0.0085) and number cases through the box (*P* = 0.6388) of light/dark box (LDB); **(D)** Immobility time of tail suspension test (TST) (*P* = 0.3973); **(E)** Immobility time of forced swimming test (FST) (*P* = 0.5834). **(F–J)** The behavioral experimental results of rutin were administered for 3 consecutive days. **(F)** Total distance for OFT (*P* = 0.5083); **(G)** OT% (*P* = 0.1738) and OE% (*P* = 0.0690) for EPM; **(H)** Time spent in the light box (*P* = 0.0366) and number cases through the box (*P* = 0.0927) of LDB; **(I)** Immobility time of TST (*P* = 0.1685); **(J)** Immobility time of FST (*P* = 0.0025). **(K–O)** The behavioral experimental results of rutin were administered for 7 consecutive days. **(K)** Total distance for OFT (*P* = 0.5821); **(L)** OT% (*P* = 0.0240) and OE% (*P* = 0.0202) for EPM; **(M)** Time spent in the light box (*P* = 0.1102) and number cases through the box (*P* = 0.0479) of LDB; **(N)** Immobility time of TST (*P* = 0.0387); **(O)** Immobility time of FST (*P* = 0.3970). The results are shown as Mean ± SD. n = 10/group. Ordinary one-way ANOVA and Tukey’s multiple comparisons test were used when the data were normally distributed and satisfied homogeneity of variance. Kruskal-Wallis test and Dunn’s multiple comparisons test were used when the data were not normally distributed. *P < 0.05.

The behavioral results of 1 day administration showed that all three dose groups of rutin had no significant effect on total distance and immobility time in mice (Figures 4A, D, E). However, Rutin M significantly increased OT% and OE% (Figure 4B) in EPM and increased the time spent in the light box in LDB (Figure 4C). Rutin administration for 3 consecutive days had no effect on the results of OFT, EPM and TST (Figures 4F, G, I). However, Rutin M increased the time spent in the light box in LDB than the control group (Figure 4H); Rutin L significantly decreased the immobility time in FST (Figure 4J). Rutin M and Rutin L produced significant results in anxiolytic-like and antidepressant-like behavioral tests after 1 and 3 days of continuous administration. Then the 7 days administration behavioral experiments were continued for both doses. Compared to the control group, Rutin L could significantly increase OT% and OE% (Figure 4L) in EPM, reduced immobility time in TST (Figure 4N); Rutin M could increase the number of cases through the box in LDB (Figure 4M). There were no significant differences in the results of both OFT and FST, but the fluoxetine and Rutin L could reduce the immobility time of mice (Figures 4K, O). We found that 12.5 mg/kg of rutin reduced the immobility time of mice at TST and FST, so we chose this dose for our study in PMDD-depressed subtype model rats.

### 3.3 Rutin alleviates depressed mood, memory impairment and social impairment in PMDD-depressed subtype rats

**FIGURE 5:**
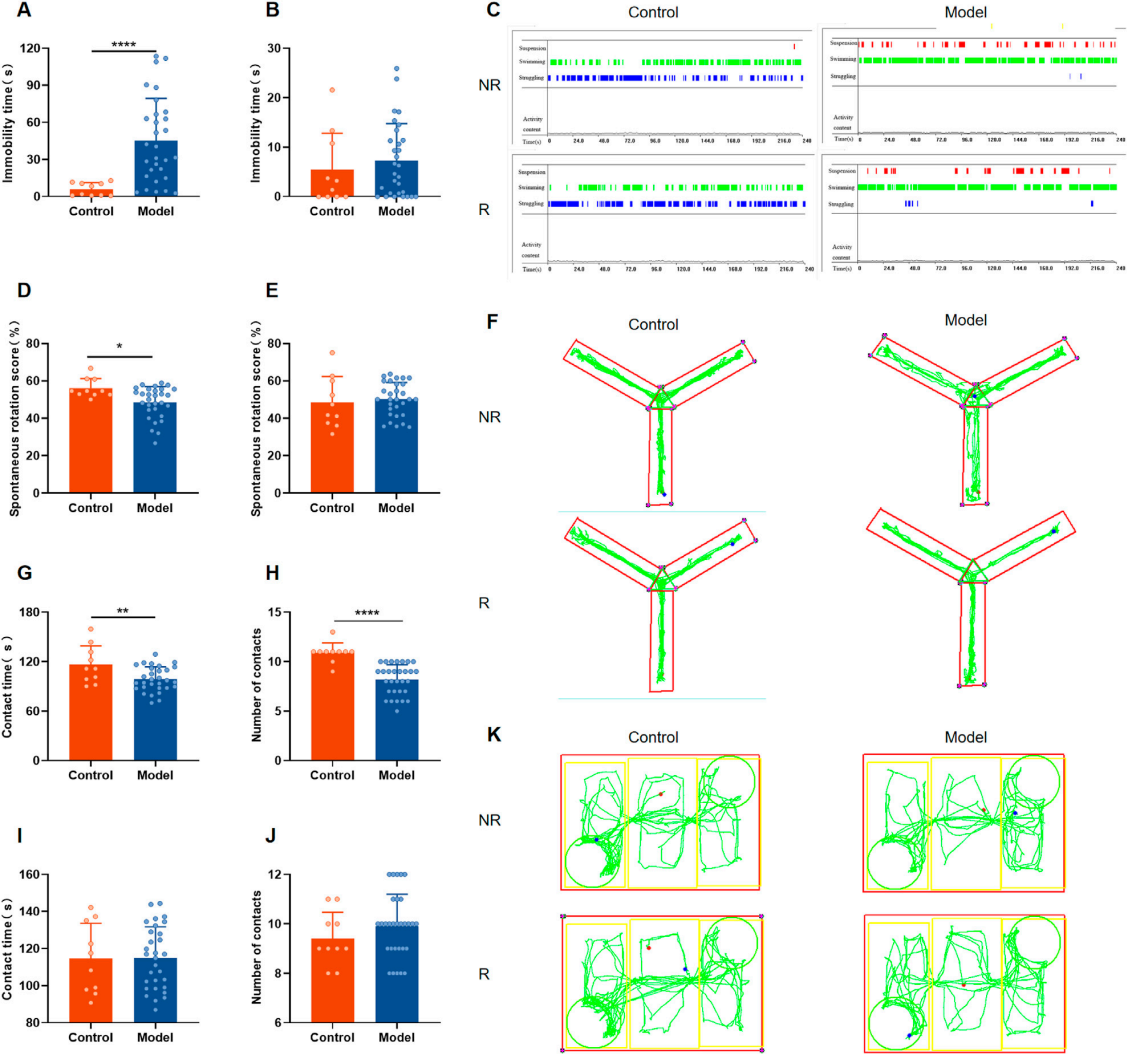
Behavioral results of rats in each group before administration. **(A)** Forced swimming test (FST) immobility time in non-receptive phase (NR) (*P* < 0.0001); **(B)** FST immobility time in receptive phase (R) (*P* = 0.3913); **(C)** representative time distribution of animals in FST; **(D)** Y-maze spontaneous rotation score in NR phase (*P* = 0.0212); **(E)** Y-maze spontaneous rotation score in R phase (*P* = 0.6613); **(F)** representative trajectory diagram of animals in Y-maze; social interaction testing (SIT) in NR phase **(G)** contact time (*P* = 0.0080), **(H)** number of contacts (*P* < 0.0001); SIT in R phase **(I)** contact time (*P* = 0.9632), **(J)** number of contacts (*P* = 0.2989); **(K)** representative trajectory diagram of animals in SIT. The results are shown as Mean ± SD. Control group: n = 10; Model group: n = 30. Parametric tests and unpaired t-tests are used when the data were normally distributed and satisfied homogeneity of variance. Nonparametric tests and Mann-Whitney tests are used when the data are not normally distributed. *P < 0.05, **P < 0.01, ****P < 0.0001.

**FIGURE 6:**
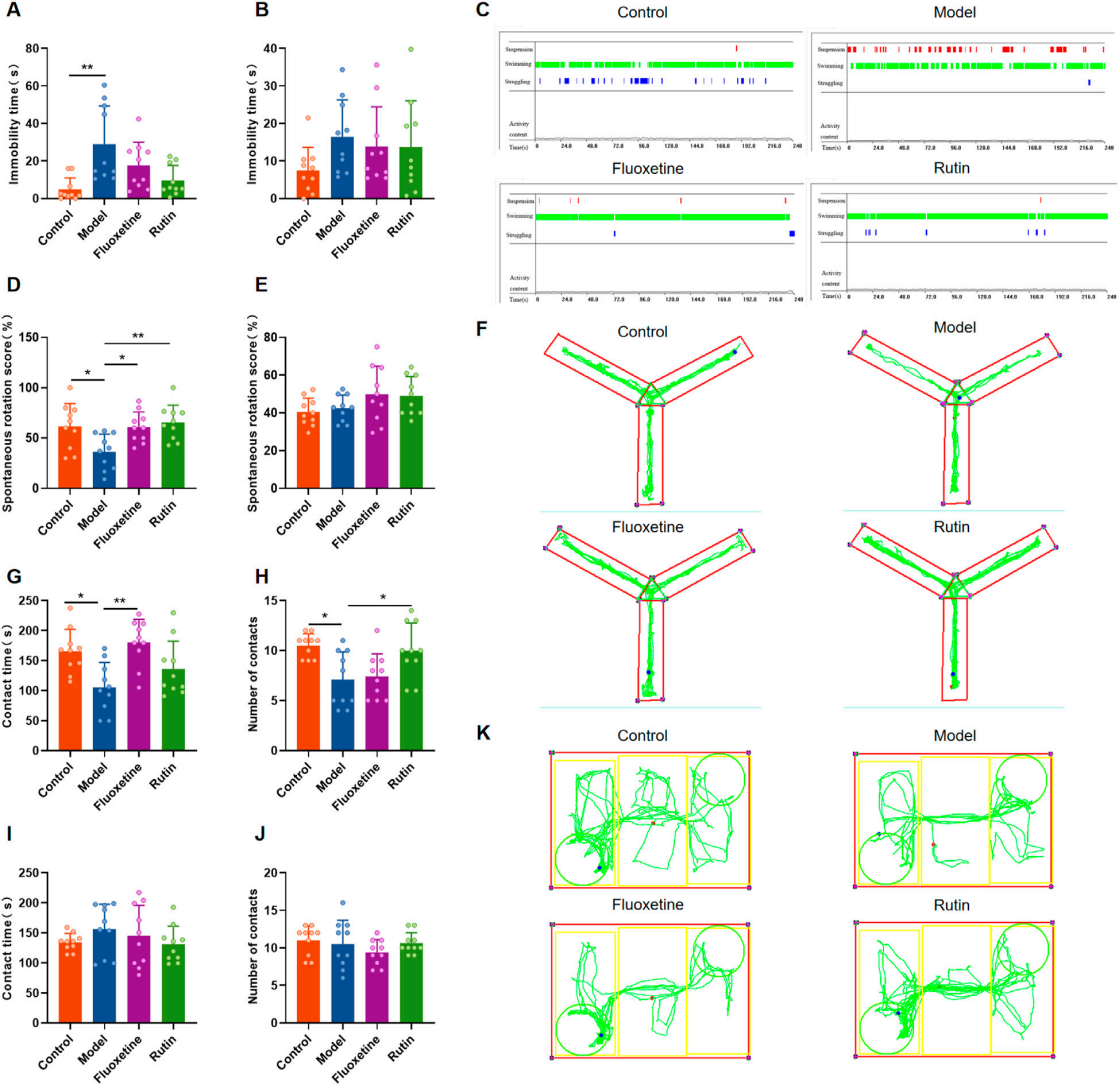
Behavioral results of each group of rats after drug administration. **(A)** Forced swimming test (FST) immobility time in non-receptive phase (NR) (*P* = 0.0013); **(B)** FST immobility time in receptive phase (R) (*P* = 0.1837); **(C)** representative time distribution of animals in FST; **(D)** Y-maze spontaneous rotation score in NR phase (*P* = 0.0046); **(E)** Y-maze spontaneous rotation score in R phase (*P* = 0.1420); **(F)** representative trajectory diagram of animals in Y-maze; social interaction testing (SIT) in NR phase **(G)** contact time (*P* = 0.0012), **(H)** number of contacts (*P* = 0.0029); SIT in R phase **(I)** contact time (*P* = 0.4381), **(J)** number of contacts (*P* = 0.4103); **(K)** representative trajectory diagram of animals in SIT. The results are shown as Mean ± SD. n = 10/group. Ordinary one-way ANOVA and Tukey’s multiple comparisons test were used when the data were normally distributed and satisfied homogeneity of variance. Brown-Forsythe and Welch ANOVA tests and Dunnett’s T3 multiple comparisons test were used when the data were normally distributed but did not satisfy homogeneity of variance. Kruskal–Wallis test and Dunn’s multiple comparisons test were used when the data were not normally distributed. *P < 0.05, **P < 0.01.

According to the studies, Rutin L (12.5 mg/kg) considerably reduced depressive-like behavior in mice. According to the body surface area drug equivalent conversion method, the amount of rutin given to the rat group was 8.65 mg/kg. To investigate the pharmacological effects of rutin on PMDD-depressed subtype rats, behavioral experiments were performed before and after administration, and the results are shown in Figures 5, 6. Prior to the administration of the drug, the model rats demonstrated an increase in immobility time (Figure 5A), a decrease in spontaneous rotation score (Figure 5D), and a significant decrease in the contact time and number of contacts with unfamiliar rats (Figures 5G, H) during the NR phase as compared to the control group. However, there were no significant difference in the behavioral data of the two groups of rats in the R phase (Figures 5B, E, I, J). And the results were confirmed by the different locomotion trajectories of the two groups of rats in the NR and R phases (Figures 5C, F, K), which is consistent with the characteristics of PMDD: “appearing before menstruation and disappearing after menstruation.” Therefore, the model was successfully prepared. The model rats were randomly divided into the model, fluoxetine, and rutin group, and behavioral experiments were conducted after two estrous cycles of drug administration. The results revealed that, compared to the control group, rats in the model group had increased immobility time (Figure 6A), decreased spontaneous rotation score (Figure 6D), and significantly decreased contact time and number of contacts with unfamiliar rats (Figures 6G, H) during the NR phase. Compared to the model group, rutin group rats showed a significant increase in spontaneous rotation score (Figure 6D), and an increase in the number of contacts with unfamiliar rats (Figure 6H); fluoxetine group showed an increase in spontaneous rotation score and contact time with unfamiliar rat (Figures 6D, G) during the NR phase. Compared with the model group, there was no significant difference in the immobility time of the rutin group, however it can be seen that there was a decreasing trend (Figure 6A). The results were also illustrated by the different locomotion trajectories of the four groups of rats during the NR phase (Figures 6C, F, K). And there was no difference between groups in R phase (Figures 6B, E, I, J). In conclusion, rutin alleviated depressed mood, memory impairment and social impairment in PMDD-depressed subtype rats.

### 3.4 Rutin alleviates hippocampal neuronal damage and neurotransmitter content changes in PMDD-depressed subtype rats

**FIGURE 7:**
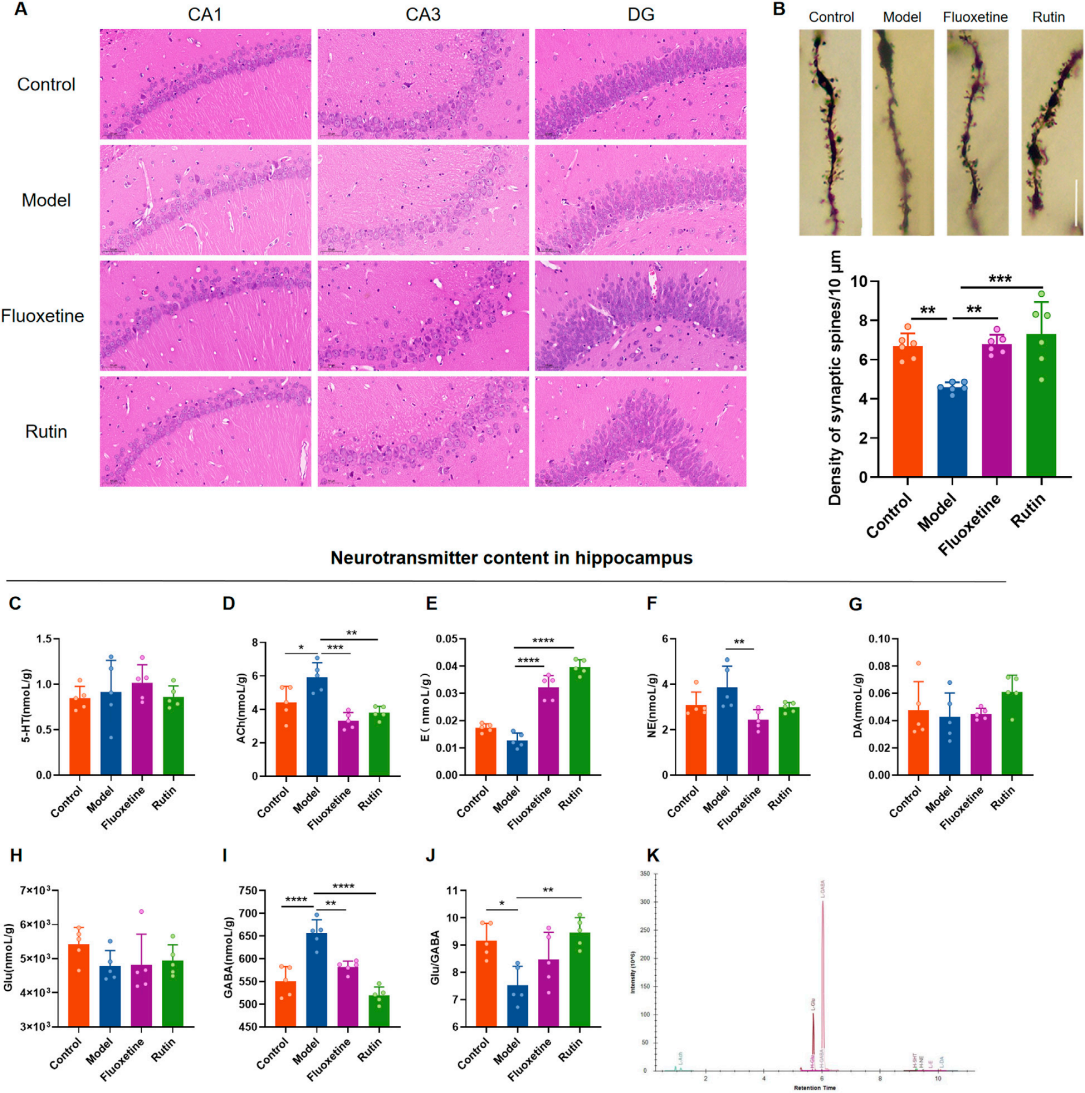
Rutin alleviates hippocampal neuronal damage and neurotransmitter content changes in PMDD-depressed subtype rats. **(A)** Comparison of hippocampal neuron cell morphology in each group of rats (HE, ×400, scale bar = 500 µm). **(B)** Comparison of dendritic spine densities in the NR phase of rats in each group (n = 6/group) (*P* = 0.0003). The scale bar represents 10 µm. **(C)** comparison of serotonin (5-HT) content (*P* = 0.6153); **(D)** comparison of acetylcholine (Ach) content (*P* = 0.0002); **(E)** comparison of epinephrine **(E)** content (*P* < 0.0001); **(F)** comparison of norepinephrine (NE) content (*P* = 0.0177); **(G)** comparison of dopamine (DA) content (*P* = 0.2696); **(H)** comparison of glutamate (Glu) content (*P* = 0.1848); **(I)** comparison of gamma-aminobutyric acid (GABA) content (*P* < 0.0001); **(J)** Glu/GABA ratio (*P* = 0.0033); **(K)** sample extraction ion chromatogram. The results are shown as Mean ± SD. n = 5/group. Ordinary one-way ANOVA and Tukey’s multiple comparisons test were used when the data were normally distributed and satisfied homogeneity of variance. Kruskal–Wallis test and Dunn’s multiple comparisons test were used when the data were not normally distributed. *P < 0.05, **P < 0.01, ***P < 0.001, ****P < 0.0001.

HE staining results showed that the hippocampal neuron cells in the control group were regular in morphology, neatly arranged, with clear cell membranes and nuclei. In the model group, the number of hippocampal neuron cells was relatively reduced, the arrangement was disorganized, the cell morphology was irregular, and the cell membrane and nucleus were unclear. Administration of fluoxetine and rutin significantly improved hippocampal neuronal damage (Figure 7A). Golgi staining revealed that the dendritic spine densities in the hippocampus of rats in the model group were significantly decreased compared to the control group. At the same time, the rutin and fluoxetine administration significantly increased the dendritic spine densities (Figure 7B). Except for the model group, each group’s dendritic morphology analysis revealed that most of the dendritic spines in the other three groups were mature spines (mushroom and short thick type). It was speculated that the injury of hippocampal dendrites in model rats manifested as reduced spine density, and rutin treatment could increase dendritic spine density.

To investigate the effect of rutin on neurotransmitter content in rat hippocampus, UHPLC-MS/MS target metabolomics was performed (Figure 7K). Acetylcholine and GABA contents in the hippocampus were significantly higher in the model group than in the control group (Figures 7D, I), and the Glu/GABA ratio was lower (Figure 7J). Acetylcholine, norepinephrine and GABA contents were decreased in the fluoxetine group compared with the model group (Figures 7D, F, I), and epinephrine contents were increased (Figure 7E). Compared to the model group, acetylcholine and GABA contents were reduced and significantly different in the rutin group (Figures 7D, I), and epinephrine content and Glu/GABA ratio were significantly higher (Figures 7E, J). There were no significant differences in 5-HT, DA and Glu contents in the hippocampus of all four groups of rats (Figures 7C, G, H).

### 3.5 Rutin does not affect the relative mRNA expression of GABA_A_ receptor subunits in rat hippocampus

**FIGURE 8:**
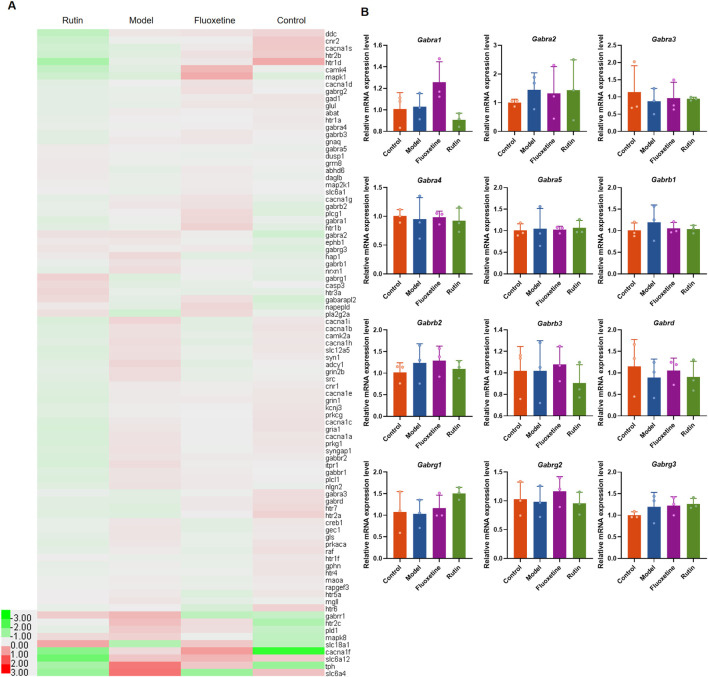
Rutin does not affect the relative mRNA expression of GABA_A_ receptor subunits in rat hippocampus. **(A)** Heatmap of PMDD-related genes. **(B)** Relative mRNA expression of GABA_A_ receptor subunits: GABA_A_ receptors α1 (*GABRA1, P* = 0.0779), α2 (*GABRA2, P* = 0.9006), α3 (*GABRA3, P* = 0.9006), α4 (*GABRA4, P* = 0.9749), α5 (*GABRA5, P* = 0.8395), β1 (*GABRB1, P* = 0.7700), β2 (*GABRB2, P* = 0.6694), β3 (*GABRB3, P* = 0.7989), δ(*GABRD, P* = 0.8738), γ1 (*GABRG1, P* = 0.2916), γ2 (*GABRG2, P* = 0.7577), and γ3 (*GABRG3, P* = 0.4139). The results are shown as Mean ± SD. n = 3/group. Ordinary one-way ANOVA and Tukey’s multiple comparisons test were used when the data were normally distributed and satisfied homogeneity of variance. Kruskal-Wallis test and Dunn’s multiple comparisons test were used when the data were not normally distributed.

Finally, we examined the relative mRNA expression of some subunits of the GABA_A_ receptor (Figure 8A). The results showed that the hippocampus of rats in the rutin group encoded GABA_A_ receptors α1 (*GABRA1*), α2 (*GABRA2*), α3 (*GABRA3*), α4 (*GABRA4*), α5 (*GABRA5*), β1 (*GABRB1*), β2 (*GABRB2*), β3 (*GABRB3*), δ(*GABRD*), γ1 (*GABRG1*), γ2 (*GABRG2*), and γ3 (*GABRG3*) genes were expressed with no significant changes (Figure 8B). Rutin may alleviate the depressive-like behavior of PMDD-depressed subtype rats by binding to GABA_A_ receptors rather than altering their relative mRNA expression.

## 4 Discussion

In this study we found that rutin binds to GABA_A_ receptor benzodiazepine sites and inhibits chloride ion inward flow. Rutin alleviated depressed mood, memory impairment, and social impairment in PMDD-depressed subtype rats, improved hippocampal neuronal damage, and reduced GABA and ACh levels. Thus rutin may exert therapeutic effects in PMDD-depressed subtype rats through the above pathways. The results of this study provide experimental and scientific basis for the development of new drugs for PMDD.

The aim of our study was to find novel drugs targeting GABA_A_ receptor to treat PMDD. Benzodiazepines, a commonly used anxiolytic, bind to GABA_A_ receptor benzodiazepine sites to increase the opening of chloride ion passage, leading to hyperpolarization of neuronal cell membranes. Subsequently, benzodiazepines enhance GABA-mediated neuronal inhibition, resulting in sedative, hypnotic and anxiolytic effects (Orzelska-Górka et al., 2020). In this experiment we found that rutin competitively binds GABA_A_ receptor with [H^3^]-flunitrazepam (Chhatwal et al., 2005). In other studies, the anxiolytic-like effects induced by intraperitoneal administration of rutin (Hernandez-Leon et al., 2017) and the anticonvulsant effects induced by ventricular injection of rutin (Nassiri-Asl et al., 2008) were eliminated by flumazenil (benzodiazepine site antagonist). This suggested that rutin does bind to the benzodiazepine site of the GABA_A_ receptor. But it was found by whole-cell membrane clamp that rutin exerts the same inhibitory effect on chloride inward flow as the GABA_A_ receptor antagonist bicuculline (Masiulis et al., 2019). Whereas the anxiolytic effect induced by rutin injection into the amygdala was abolished by picrotoxin (chloride channel GABA_A_ antagonist) (Hernandez-Leon et al., 2017), Nassiri-Asl also mentioned that rutin may positively metabolize and modulate GABA_A_ receptors (Nassiri-Asl et al., 2008). This is not consistent with our results. We hypothesize that rutin exerts both actions may be dose dependent, which requires further study. Although some studies have found altered protein and mRNA expression of partial subunits of GABA_A_ receptors in brain tissues such as the hippocampus of PMDD rats (Zhang et al., 2023; Gao et al., 2022a; Wei et al., 2020), which is not consistent with the present study. A review of the literature also did not reveal any studies related to rutin and GABAA receptor subunit expression. Thus, we hypothesized that rutin may exert therapeutic effects in PMDD-depressed subtype model rats by binding to GABA_A_ receptor benzodiazepine sites and inhibiting chloride inward flow, rather than affecting the relative expression of GABA_A_ receptor subunit mRNA.

Hippocampal neurons are important structures in the brain responsible for memory and spatial navigation. Dendritic spines are synaptic contact points between neuronal dendrites and the axon terminals of neighboring neurons and are important for neuronal signaling. Recent studies have found apoptosis and damage to hippocampal neurons in PMDD rats (Wei et al., 2023; Li et al., 2023), and chronic restraint stress leads to a decrease in dendritic spine density (Shen et al., 2021). We found that rutin ameliorated hippocampal neuronal damage and increased dendritic spine density in PMDD-depressed subtype rats. This is consistent with the results that rutin exerts a protective effect against chronic stress-induced hippocampal neuronal loss (Parashar et al., 2017). It has also been found that rutin protects against copper-induced neuronal degeneration and cortical perforation in rats through antioxidant and anti-inflammatory properties (Arowoogun et al., 2021). In addition, abnormally phosphorylated forms of tau aggregate and accumulate into neurofibrillary tangles, leading to synapse loss, neuroinflammation, and neurodegeneration. Rutin reduced pathological tau levels, suppressed gliosis and neuroinflammation by downregulating NF-κB pathway, prevented microglial synapse engulfment, and rescued synapse loss in mouse brains, resulting in a significant improvement of cognition (Sun et al., 2021). Rutin also attenuates neuronal loss in cerebral ischemia/reperfusion injury rats through estrogen receptor-mediated BDNF-TrκB and NGF-TrκA signaling pathways (Liu S. et al., 2018). The above shows the pathway of rutin in treating hippocampal neuronal damage, which provides potential targets for subsequent studies of rutin to improve the morphology of hippocampal neurons in PMDD-depressed subtype rats.

Normally, glutamate decarboxylase can decarboxylate the excitatory neurotransmitter glutamate (Glu) to GABA (Scotti-Muzzi et al., 2021), and their levels *in vivo* remain in dynamic balance. When GABA binds to the GABA_A_ receptor on the postsynaptic membrane, Ca^2+^ inward flow is restricted, Cl^−^ inward flow, resulting in reduced neuronal excitability (Li and Xu, 2008). When the central nervous system is affected, the Glu/GABA ratio is commonly used to assess its excitatory or inhibitory state (Wang et al., 2020). Sun (2020) found elevated GABA levels in the rats' hippocampus with the PMDD anxiety subtype. Gao et al. (2014) found a significant decrease in the Glu/GABA ratio in the hippocampal extracellular fluid of rats with the PMS depression subtype. Elevated GABA levels and decreased Glu levels, Glu/GABA ratio were also found in rats with electroshock-induced depressive learning memory disorder (Luo et al., 2011). The above findings are consistent with our findings, suggesting that rutin may also be able to treat the PMDD-depressed subtype model rats by decreasing GABA content and increasing the Glu/GABA ratio in the hippocampus. In addition, we found elevated levels of acetylcholine in the hippocampal brain region of model rats, and rutin intervention reduced acetylcholine levels, which is also an innovative finding. There are fewer studies related to PMDD and acetylcholine. However, the research on depression has shown that depressed people have higher acetylcholine levels and an overactive cholinergic system. The antidepressant effect can be produced by antagonizing nicotinic or muscarinic acetylcholine receptors to reduce cholinergic signaling (Mineur et al., 2022). Therefore, we hypothesized that rutin might treat PMDD-depressed subtype model rats by decreasing acetylcholine levels in the hippocampus and inhibiting overactivation of the cholinergic system.

This research offers a scientific rationale for the clinical use of rutin in treating PMDD and an experimental foundation for creating innovative drugs. However, this study lacks long-term effects and dependence testing of rutin, and is shallow in terms of mechanism. The mechanism of action of rutin in treating PMDD-depressed subtype model rats can be explored in depth around the findings in this study. In addition, the poor solubility and bioavailability of rutin limit its application. Therefore, developing new dosage forms of rutin is also a hot topic of current research. It has been found that rutin solid lipid nanoparticles can effectively cross the blood-brain barrier and target tumors to act (Pandian et al., 2021). Pan et al. (2019) found that rutin sodium significantly improved its water solubility and bioavailability and treated Alzheimer’s disease by acting on microglia. Follow-up studies will be centered on the present findings to deeply explore the interrelationships of the mechanism of action of rutin in the treatment of PMDD, and to integrate the multidisciplinary fields to create a new drug delivery system, which will provide an optimal choice for the development of new drugs and the clinical treatment of PMDD.

## Data Availability

The original contributions presented in the study are publicly available. This data can be found here: https://doi.org/10.6084/m9.figshare.28148204.v1.

## References

[B1] Arowoogun J. Akanni O. O. Adefisan A. O. Owumi S. E. Tijani A. S. Adaramoye O. A. (2021). Rutin ameliorates copper sulfate‐induced brain damage via antioxidative and anti‐inflammatory activities in rats. J. Biochem. Mol. Toxicol. 35 (1), e22623. 10.1002/jbt.22623 32881150

[B2] Bäckström T. Haage D. Löfgren M. Johansson I. M. Strömberg J. Nyberg S. (2011). Paradoxical effects of GABA-A modulators may explain sex steroid induced negative mood symptoms in some persons. Neuroscience 191, 46–54. 10.1016/j.neuroscience.2011.03.061 21600269

[B3] Barki M. Xue H. (2022). GABRB2, a key player in neuropsychiatric disorders and beyond. Gene 809, 146021. 10.1016/j.gene.2021.146021 34673206

[B4] Bian X. Zhang Y. Huang B. Wang X. Wang G. Zhu Y. (2019). Natural product incarvillateine aggravates epileptic seizures by inhibiting GABA(A) currents. Eur. J. Pharmacol. 858, 172496. 10.1016/j.ejphar.2019.172496 31242440

[B5] Borbély É. Hajna Z. Nabi L. Scheich B. Tékus V. László K. (2017). Hemokinin-1 mediates anxiolytic and anti-depressant-like actions in mice. Brain, Behav. Immun. 59, 219–232. 10.1016/j.bbi.2016.09.004 27621226

[B6] Budzynska B. Faggio C. Kruk-Slomka M. Samec D. Nabavi S. F. Sureda A. (2019). Rutin as neuroprotective agent: from bench to bedside. Curr. Med. Chem. 26 (27), 5152–5164. 10.2174/0929867324666171003114154 28971760

[B7] Chebib S. Schwab W. (2021). Microscale thermophoresis reveals oxidized glutathione as high-affinity ligand of mal d 1. Foods 10 (11), 2771. 10.3390/foods10112771 34829051 PMC8618550

[B8] Chhatwal J. P. Myers K. M. Ressler K. J. Davis M. (2005). Regulation of gephyrin and GABAA receptor binding within the amygdala after fear acquisition and extinction. J. Neurosci. 25 (2), 502–506. 10.1523/JNEUROSCI.3301-04.2005 15647495 PMC6725488

[B9] Daina A. Michielin O. Zoete V. (2017). SwissADME: a free web tool to evaluate pharmacokinetics, drug-likeness and medicinal chemistry friendliness of small molecules. Sci. Rep. 7 (1), 42717. 10.1038/srep42717 28256516 PMC5335600

[B10] Du F. (2019). Golgi‐cox staining of neuronal dendrites and dendritic spines with FD rapid GolgiStain™ kit. Current Protoc. Neurosci. 88 (1), e69. 10.1002/cpns.69 31216393

[B11] Ferreira R. S. Teles-Souza J. Dos Santos Souza C. Pereira É. P. L. de Araújo F. M. Da Silva A. B. (2021). Rutin improves glutamate uptake and inhibits glutamate excitotoxicity in rat brain slices. Mol. Biol. Rep. 48 (2), 1475–1483. 10.1007/s11033-021-06145-y 33492574

[B12] Foudah A. I. Alqarni M. H. Alam A. Devi S. Salkini M. A. Alam P. (2022). Rutin improves anxiety and reserpine-induced depression in rats. Molecules 27 (21), 7313. 10.3390/molecules27217313 36364141 PMC9654015

[B13] Friesner R. A. Murphy R. B. Repasky M. P. Frye L. L. Greenwood J. R. Halgren T. A. (2006). Extra precision glide: docking and scoring incorporating a model of hydrophobic enclosure for Protein−Ligand complexes. J. Med. Chem. 49 (21), 6177–6196. 10.1021/jm051256o 17034125

[B14] Gao M. Zhang H. Gao Z. Sun Y. Xu G. Wei F. (2022a). Resident intruder paradigm-induced PMDD rat model of premenstrual irritability: behavioral phenotypes, drug intervention, and biomarkers. Aging (Albany NY) 14, 9210–9220. 10.18632/aging.204402 36441533 PMC9740374

[B15] Gao M. Zhang H. Sun Y. Gao Z. Sun C. Wei F. (2022b). Gabrb2 knock-out mice exhibit double-directed PMDD-like symptoms: GABAAR subunits, neurotransmitter metabolism disruption, and allopregnanolone binding. Aging (Albany, NY.) 14 (20), 8437–8447. 10.18632/aging.204351 36287173 PMC9648806

[B16] Gao X. Sun P. Qiao M. Wei S. Xue L. Zhang H. (2014). Shu-Yu capsule, a Traditional Chinese Medicine formulation, attenuates premenstrual syndrome depression induced by chronic stress constraint. Mol. Med. Rep. 10 (6), 2942–2948. 10.3892/mmr.2014.2599 25270424

[B17] Geng X. Wang X. Liu K. Xing Y. Xu J. Li Z. (2024). ShuYu capsule alleviates emotional and physical symptoms of premenstrual dysphoric disorder: impact on ALLO decline and GABAA receptor δ subunit in the PAG area. Phytomedicine 130, 155549. 10.1016/j.phymed.2024.155549 38810551

[B18] Green N. F. O. Maniam J. Riese J. Morris M. J. Voineagu I. (2021). Transcriptomic signature of early life stress in male rat prefrontal cortex. Neurobiol. Stress 14, 100316. 10.1016/j.ynstr.2021.100316 33796639 PMC7995657

[B19] Hantsoo L. Payne J. L. (2023). Towards understanding the biology of premenstrual dysphoric disorder: from genes to GABA. Neurosci. & Biobehav. Rev. 149, 105168. 10.1016/j.neubiorev.2023.105168 37059403 PMC10176022

[B20] Hernandez-Leon A. González-Trujano M. E. Fernández-Guasti A. (2017). The anxiolytic-like effect of rutin in rats involves GABAA receptors in the basolateral amygdala. Behav. Pharmacol. 28 (4), 303–312. 10.1097/FBP.0000000000000290 28145981

[B21] Huang L. Zhang C. (2021). Microscale thermophoresis (MST) to detect the interaction between purified protein and small molecule. Methods Mol. Biol. 2213, 187–193. 10.1007/978-1-0716-0954-5_17 33270204

[B22] Hubscher C. H. Brooks D. L. Johnson J. R. (2009). A quantitative method for assessing stages of the rat estrous cycle. Biotech. Histochem. 80 (2), 79–87. 10.1080/10520290500138422 16195173

[B23] Jeong H. Ham B. Yeo H. B. Jung I. Joe S. (2012). Gray matter abnormalities in patients with premenstrual dysphoric disorder: an optimized voxel-based morphometry. J. Affect. Disord. 140 (3), 260–267. 10.1016/j.jad.2012.02.010 22381950

[B24] Ko Y. Kim S. Lee S. Jang C. (2020). Flavonoids as therapeutic candidates for emotional disorders such as anxiety and depression. Arch. Pharm. Res. 43 (11), 1128–1143. 10.1007/s12272-020-01292-5 33225387

[B25] Lamtai M. Azirar S. Zghari O. Ouakki S. El Hessni A. Mesfioui A. (2021). Melatonin ameliorates cadmium-induced affective and cognitive impairments and hippocampal oxidative stress in rat. Biol. Trace Elem. Res. 199 (4), 1445–1455. 10.1007/s12011-020-02247-z 32613486

[B26] Lanza Di Scalea T. Pearlstein T. (2019). Premenstrual dysphoric disorder. Med. Clin. N. Am. 103 (4), 613–628. 10.1016/j.mcna.2019.02.007 31078196

[B27] Laskowski R. A. Swindells M. B. (2011). LigPlot+: multiple ligand–protein interaction diagrams for drug discovery. J. Chem. Inf. Model. 51 (10), 2778–2786. 10.1021/ci200227u 21919503

[B28] Li K. Xu E. (2008). The role and the mechanism of γ-aminobutyric acid during central nervous system development. Neurosci. Bull. 24 (3), 195–200. 10.1007/s12264-008-0109-3 18500393 PMC5552538

[B29] Li S. Mu X. Ma S. Li X. Gao J. Liu X. (2023). Xiangshao Granules reduce the aggressive behavior and hippocampal injury of premenstrual irritability in rats by regulating JIK/JNK/p38 signal pathway. J. Ethnopharmacol. 305, 116061. 10.1016/j.jep.2022.116061 36577489

[B30] Li Y. Luo Y. Xu W. Ge J. Cherasse Y. Wang Y. (2021). Ventral pallidal GABAergic neurons control wakefulness associated with motivation through the ventral tegmental pathway. Mol. Psychiatr. 26 (7), 2912–2928. 10.1038/s41380-020-00906-0 PMC8505244 33057171

[B31] Liang X. Xue Z. Zheng Y. Li S. Zhou L. Cao L. (2023). Selenium supplementation enhanced the expression of selenoproteins in hippocampus and played a neuroprotective role in LPS-induced neuroinflammation. Int. J. Biol. Macromol. 234, 123740. 10.1016/j.ijbiomac.2023.123740 36806773

[B32] Lin Y. Peng W. Shih M. Cherng J. (2021). Anxiolytic effect of an extract of Salvia miltiorrhiza Bunge (Danshen) in mice. J. Ethnopharmacol. 264, 113285. 10.1016/j.jep.2020.113285 32827660

[B33] Liu H. Zhang T. A. Zhang W. Y. Huang S. R. Hu Y. Sun J. (2023). Rhein attenuates cerebral ischemia-reperfusion injury via inhibition of ferroptosis through NRF2/SLC7A11/GPX4 pathway. Exp. Neurol. 369, 114541. 10.1016/j.expneurol.2023.114541 37714424

[B34] Liu R. Xian Y. Liu S. Yu F. Mu H. Sun K. (2018). Development, validation and comparison of surrogate matrix and surrogate analyte approaches with UHPLC-MS/MS to simultaneously quantify dopamine, serotonin and γ-aminobutyric acid in four rat brain regions. Biomed. Chromatogr. 32 (9), e4276. 10.1002/bmc.4276 29727024

[B35] Liu S. Fan M. Xu J. Yang L. Qi C. Xia Q. (2022). Exosomes derived from bone-marrow mesenchymal stem cells alleviate cognitive decline in AD-like mice by improving BDNF-related neuropathology. J. Neuroinflamm. 19 (1), 35. 10.1186/s12974-022-02393-2 PMC8822863 35130907

[B36] Liu S. Xu S. Wang Z. Guo Y. Pan W. Shen Z. (2018). Anti-Depressant-like effect of sinomenine on chronic unpredictable mild stress-induced depression in a mouse model. Med. Sci. Monit. 24, 7646–7653. 10.12659/MSM.908422 30362468 PMC6215386

[B37] Liu H. Zhong L. Zhang Y. Liu X. Li J. (2018). Rutin attenuates cerebral ischemia-reperfusion injury in ovariectomized rats via estrogen-receptor-mediated BDNF-TrkB and NGF-TrkA signaling. Biochem. Cell Biol. 96 (5), 672–681. 10.1139/bcb-2017-0209 29420916

[B38] Livak K. J. Schmittgen T. D. (2001). Analysis of relative gene expression data using real-time quantitative PCR and the 2(-Delta Delta C(T)) Method. Methods 25 (4), 402–408. 10.1006/meth.2001.1262 11846609

[B39] Luo J. Min S. Wei K. Li P. Dong J. Liu Y. (2011). Propofol protects against impairment of learning-memory and imbalance of hippocampal Glu/GABA induced by electroconvulsive shock in depressed rats. J. Anesth. 25 (5), 657–665. 10.1007/s00540-011-1199-z 21769668

[B40] Martinez P. E. Rubinow D. R. Nieman L. K. Koziol D. E. Morrow A. L. Schiller C. E. (2016). 5α-Reductase inhibition prevents the luteal phase increase in plasma allopregnanolone levels and mitigates symptoms in women with premenstrual dysphoric disorder. Neuropsychopharmacology 41 (4), 1093–1102. 10.1038/npp.2015.246 26272051 PMC4748434

[B41] Masiulis S. Desai R. Uchański T. Serna Martin I. Laverty D. Karia D. (2019). GABAA receptor signalling mechanisms revealed by structural pharmacology. Nat. Lond. 565 (7740), 454–459. 10.1038/s41586-018-0832-5 30602790 PMC6370056

[B42] Mckernan R. M. Whiting P. J. (1996). Which GABAA-receptor subtypes really occur in the brain? Trends Neurosci. 19 (4), 139–143. 10.1016/s0166-2236(96)80023-3 8658597

[B43] Middendorp S. J. Maldifassi M. C. Baur R. Sigel E. (2015). Positive modulation of synaptic and extrasynaptic GABAA receptors by an antagonist of the high affinity benzodiazepine binding site. Neuropharmacology 95, 459–467. 10.1016/j.neuropharm.2015.04.027 25963418

[B44] Mineur Y. S. Mose T. N. Vanopdenbosch L. Etherington I. M. Ogbejesi C. Islam A. (2022). Hippocampal acetylcholine modulates stress-related behaviors independent of specific cholinergic inputs. Mol. Psychiatr. 27, 1829–1838. 10.1038/s41380-021-01404-7 PMC9106825 34997190

[B45] Naguy A. El-Sheshai A. Thiguti S. H. Alamiri B. (2022). Psychopharmacotherapy of premenstrual dysphoric disorder-new vistas. Psychopharmacol. Bull. 52 (3), 81–83. 35815174 10.64719/pb.4447 PMC9235312

[B46] Nassiri-Asl M. Shariati-Rad S. Zamansoltani F. (2008). Anticonvulsive effects of intracerebroventricular administration of rutin in rats. Prog. Neuro-Psychopharmacology Biol. Psychiatry 32 (4), 989–993. 10.1016/j.pnpbp.2008.01.011 18262708

[B47] Okojie A. K. Oyekunle O. A. (2014). Depo-Provera effects on Wistar rat performance in the Y-maze. Metab. Brain Dis. 29 (2), 529–531. 10.1007/s11011-013-9460-9 24338027

[B48] Orzelska-Górka J. Bernat P. Tutka P. Listos J. Kędzierska E. Fidecka S. (2020). Modification of NO-cGMP pathway differentially affects diazepam- and flunitrazepam-induced spatial and recognition memory impairments in rodents. Neurotox. Res. 37 (4), 1036–1046. 10.1007/s12640-019-00110-1 31792805 PMC7085477

[B49] Pan R. Y. Ma J. Kong X. X. Wang X. F. Li S. S. Qi X. L. (2019). Sodium rutin ameliorates Alzheimer's disease-like pathology by enhancing microglial amyloid-β clearance. Sci. Adv. 5 (2), eaau6328. 10.1126/sciadv.aau6328 30820451 PMC6393001

[B50] Pandian S. R. K. Pavadai P. Vellaisamy S. Ravishankar V. Palanisamy P. Sundar L. M. (2021). Formulation and evaluation of rutin-loaded solid lipid nanoparticles for the treatment of brain tumor. Naunyn-Schmiedeberg's Archives Pharmacol. 394 (4), 735–749. 10.1007/s00210-020-02015-9 33156389

[B51] Parashar A. Mehta V. Udayabanu M. (2017). Rutin alleviates chronic unpredictable stress-induced behavioral alterations and hippocampal damage in mice. Neurosci. Lett. 656, 65–71. 10.1016/j.neulet.2017.04.058 28732760

[B52] Qiao M. Sun P. Wang H. Wang Y. Zhan X. Liu H. (2017). Epidemiological distribution and subtype analysis of premenstrual dysphoric disorder syndromes and symptoms based on TCM theories. Biomed. Res. Int. 2017, 4595016–4595019. 10.1155/2017/4595016 28698873 PMC5494079

[B53] Re Nappi L. Nappi R. E. (2022). Recent advances in understanding/management of premenstrual dysphoric disorder/premenstrual syndrome. Fac. Rev. 11, 11. 10.12703/r/11-11 35574174 PMC9066446

[B54] Scotti-Muzzi E. Chile T. Moreno R. Pastorello B. F. Da Costa Leite C. Henning A. (2021). ACC Glu/GABA ratio is decreased in euthymic bipolar disorder I patients: possible *in vivo* neurometabolite explanation for mood stabilization. Eur. Arch. Psych. Clin. Neurosci. 271 (3), 537–547. 10.1007/s00406-020-01096-0 31993746

[B55] Seibenhener M. L. Wooten M. C. (2015). Use of the open field maze to measure locomotor and anxiety-like behavior in mice. J. Vis. Exp. 96, e52434. 10.3791/52434 PMC4354627 25742564

[B56] Shen J. Yang L. Wei W. (2021). Role of Fto on CaMKII/CREB signaling pathway of hippocampus in depressive-like behaviors induced by chronic restraint stress mice. Behav. Brain Res. 406, 113227. 10.1016/j.bbr.2021.113227 33677012

[B57] Stiernman L. Dubol M. Comasco E. Sundstrom-Poromaa I. Boraxbekk C. J. Johansson M. (2023). Emotion-induced brain activation across the menstrual cycle in individuals with premenstrual dysphoric disorder and associations to serum levels of progesterone-derived neurosteroids. Transl. Psychiatr. 13 (1), 124. 10.1038/s41398-023-02424-3 PMC10101953 37055419

[B58] Study R. E. Barker J. L. (1981). Diazepam and (--)-pentobarbital: fluctuation analysis reveals different mechanisms for potentiation of gamma-aminobutyric acid responses in cultured central neurons. Proc. Natl. Acad. Sci. U. S. A. 78 (11), 7180–7184. 10.1073/pnas.78.11.7180 6273918 PMC349220

[B59] Sun X. Li L. Dong Q. Zhu J. Huang Y. Hou S. (2021). Rutin prevents tau pathology and neuroinflammation in a mouse model of Alzheimer’s disease. J. Neuroinflamm. 18 (1), 131. 10.1186/s12974-021-02182-3 PMC8196535 34116706

[B60] Sun Y. (2020). Study on the underlying mechanism of dsorder cause by the liver failing to conver and disperse-allopregnanolone mediated GABAARα4 subunit sensitivity in the pathogenesis of specific brain regions of premenstrual dysphoric disorder liver QiInverse. Master’s thesis. Jinan (China): Shandong University of Traditional Chinese Medicine.

[B61] Syan S. K. Minuzzi L. Smith M. Costescu D. Allega O. R. Hall G. B. C. (2018). Brain structure and function in women with comorbid bipolar and premenstrual dysphoric disorder. Front. Psychiatry. 8, 301. 10.3389/fpsyt.2017.00301 29367847 PMC5768056

[B62] Thompson S. M. (2024). Modulators of GABAA receptor-mediated inhibition in the treatment of neuropsychiatric disorders: past, present, and future. Neuropsychopharmacol. (New York, N.Y.) 49 (1), 83–95. 10.1038/s41386-023-01728-8 PMC10700661 37709943

[B63] Vogel H. W. (1986). “Benzodiazepine receptor: [3H]-flflunitrazepam binding as say,” in Drug discovery and evaluation: Pharmacology assay. Editors Vogel, H. G. Vogel H. W. (New York: Springer), 408e409.

[B64] Wang M. Li N. Jing S. Wang C. Sun J. Li H. (2020). Schisandrin B exerts hypnotic effects in PCPA-treated rats by increasing hypothalamic 5-HT and γ-aminobutyric acid levels. Exp. Ther. Med. 20 (6), 142. 10.3892/etm.2020.9271 33093880 PMC7571383

[B65] Wang Y. Wu H. Zhang S. Xiao H. Yu J. Ma Y. (2021). Catalpol ameliorates depressive-like behaviors in CUMS mice via oxidative stress-mediated NLRP3 inflammasome and neuroinflammation. Transl. Psychiatr. 11 (1), 353. 10.1038/s41398-021-01468-7 PMC8187638 34103482

[B66] Wei E. Gao A. Mu X. Qu S. Yang C. Li F. (2023). Paeonol ameliorates hippocampal neuronal damage by inhibiting GRM5/GABBR2/β-arrestin2 and activating the cAMP-PKA signaling pathway in premenstrual irritability rats. Brain Res. Bull. 205, 110830. 10.1016/j.brainresbull.2023.110830 38036272

[B67] Wei S. Geng X. Li Z. Xu K. Hu M. Wu H. (2020). A forced swim-based rat model of premenstrual depression: effects of hormonal changes and drug intervention. Aging 12 (23), 24357–24370. 10.18632/aging.202249 33229622 PMC7762461

[B68] Wei S. Sun P. Guo Y. Chen J. Wang J. Song C. (2018). Gene expression in the Hippocampus in a rat model of premenstrual dysphoric disorder after treatment with baixiangdan capsules. Front. Psychol. 9, 2065. 10.3389/fpsyg.2018.02065 30483168 PMC6242977

[B69] Wen Y. Dong Z. Liu J. Axerio-Cilies P. Du Y. Li J. (2022). Glutamate and GABA(A) receptor crosstalk mediates homeostatic regulation of neuronal excitation in the mammalian brain. Signal Transduct. Target. Ther. 7 (1), 340. 10.1038/s41392-022-01148-y 36184627 PMC9527238

[B70] Wong J. T. Malec P. A. Mabrouk O. S. Ro J. Dus M. Kennedy R. T. (2016). Benzoyl chloride derivatization with liquid chromatography–mass spectrometry for targeted metabolomics of neurochemicals in biological samples. J. Chromatogr. A 1446, 78–90. 10.1016/j.chroma.2016.04.006 27083258 PMC4845038

[B71] Xue H. Wu Z. Long X. Ullah A. Chen S. Mat W. K. (2021). Copy number variation profile-based genomic typing of premenstrual dysphoric disorder in Chinese. J. Genet. Genomics. 48 (12), 1070–1080. 10.1016/j.jgg.2021.08.012 34530168

[B72] Yue S. Yu J. Kong Y. Chen H. Mao M. Ji C. (2019). Metabolomic modulations of HepG2 cells exposed to bisphenol analogues. Environ. Int. 129, 59–67. 10.1016/j.envint.2019.05.008 31121516

[B73] Zhang H. Gao Z. Sun Y. Lu T. Wang Z. Gao D. (2023). Profiling GABA(A) receptor subunit expression in the Hippocampus of PMDD rat models based on TCM theories. Mol. Neurobiol. 60 (8), 4418–4428. 10.1007/s12035-023-03354-3 37103685

[B74] Zhou Y. Liu X. Xue J. Liu L. Liang T. Li W. (2020). Discovery of peptide boronate derivatives as histone deacetylase and proteasome dual inhibitors for overcoming bortezomib resistance of multiple myeloma. J. Med. Chem. 63 (9), 4701–4715. 10.1021/acs.jmedchem.9b02161 32267687

